# Comparative analysis of fasting effects on the cecum microbiome in three guinea pig breeds: Andina, Inti, and Peru

**DOI:** 10.3389/fmicb.2023.1283738

**Published:** 2023-12-20

**Authors:** Hugo Frias, Nilton Luis Murga Valderrama, Gary J. Flores Durand, Victor G. Cornejo, Ana C. Romani, William Bardales, G. T. Segura, Richard C. Polveiro, Dielson da S. Vieira, Eduardo M. Ramos Sanchez, Rainer M. Lopez Lapa, Jorge Luis Maicelo Quintana

**Affiliations:** ^1^Facultad de Ingeniería Zootecnista, Agronegocios y Biotecnología, Instituto de Investigación en Ganadería y Biotecnología, Universidad Nacional Toribio Rodríguez de Mendoza de Amazonas, Chachapoyas, Peru; ^2^Facultad de Ingeniería Zootecnista, Agronegocios y Biotecnología, Universidad Nacional Toribio Rodríguez de Mendoza de Amazonas, Chachapoyas, Peru; ^3^Laboratorio de Fisiología Molecular, Facultad de Ingeniería Zootecnista, Agronegocios y Biotecnología, Instituto de Investigación en Ganadería y Biotecnología, Universidad Nacional Toribio Rodríguez de Mendoza de Amazonas, Chachapoyas, Peru; ^4^Laboratorio de Enfermedades Infecciosas y Parasitarias, Facultad de Ingeniería Zootecnista, Agronegocios y Biotecnología, Instituto de Investigación en Ganadería y Biotecnología, Universidad Nacional Toribio Rodríguez de Mendoza de Amazonas, Chachapoyas, Peru; ^5^Laboratory of Bacterial Diseases, Sector of Preventive Veterinary Medicine and Public Health, Department of Veterinary, Universidade Federal de Viçosa, Viçosa, MG, Brazil; ^6^Department of Basic Medical Sciences, College of Veterinary Medicine, Purdue University, West Lafayette, IN, United States; ^7^Chemistry Department, Institute for Drug Discovery, Purdue University, West Lafayette, IN, United States; ^8^Programa de Pós-Graduação em Ciência Animal, Departamento de Zootecnia, Centro de Ciências Agrárias, Universidade Federal da Paraíba, Areia, Brazil; ^9^Facultad de Medicina, Universidad Nacional Toribio Rodríguez de Mendoza de Amazonas, Chachapoyas, Peru

**Keywords:** cecum microbiome, Guinea pig, Andina breed, Inti breed, Peru breed, fasting

## Abstract

Guinea pigs have historically been used as a food source and are also an important model for studying the human intestines. Fasting is the act of temporarily stopping the intake of food. This process can alter the microbiota of various animals. This study is the first to investigate the impact of fasting on the cecum microbiome of three guinea pig breeds. We investigated the impact of fasting on the microbiome population structure in the cecum of three guinea pig breeds. This was done by sequencing and analyzing the V4 hypervariable region of the 16S rRNA gene in bacterial communities found in cecum mucosa samples. To achieve this, we established two treatment groups (fasting and fed), for each of the three guinea pig breeds: Andina, Inti, and Peru. The study involved twenty-eight guinea pigs, which were divided into the following groups: Andina-fed (five), Andina-fasting (five), Inti-fed (four), Inti-fasting (five), Peru-fed (five), and Peru-fasting (four). The results indicated a significant difference in beta diversity between the treatment groups for the Peru breed (*P*-value = 0.049), but not for the treatment groups of the Andina and Inti breeds. The dominant phyla across all groups were Firmicutes and Bacteroidetes. We observed variations in the abundance of different taxa in the cecum microbiota when comparing the treatment groups for each breed. Additionally, there was a higher number of unique taxa observed in the fasting groups compared to the fed groups. We discovered that the genus *Victivallis* was the only one present in all fasting groups across all breeds. Despite the findings, the resilience of the gut microbiome was not challenged in all three breeds, which can lead to disruptive changes that may affect the overall maintenance of the cecum microbiome. Based on the observed differences in the treatment groups of the Peru breed, it can be suggested that fasting has a greater impact on this particular breed.

## 1 Introduction

Fasting is a process that can generate several changes in the human and animal body. Since the 1920s, it has been known that fasting has an impact on the composition of the microbiome (Benedict, 1915); however, the direct relationship is still uncertain. The process of fasting can cause a reduction in nutrient availability since the microbiome can be affected (McCue et al., 2017). During fasting, bacteria that use host-derived substrates, such as mucins, shed epithelial cells and proliferate. Still, bacteria that depend on food substrates decrease their abundance because of the food intake irruption (Ducarmon et al., 2023). Another factor associated with the microbiome change during fasting is intestinal tissue remodeling. This process is known as transient atrophy of the intestinal tissue. It is characterized by a decrease in mitosis in crypts and an increment in the rate of apoptosis and autophagy (Habold et al., 2004). For example, in rats, the mucosal mass decreases by 50% during fasting (Dunel-Erb et al., 2001).

Guinea pigs (*Cavia porcellus*) have been used for different motives such as a food source and popular pets by humans (Buela et al., 2022). Another human utility of this animal is human intestinal research (Hildebrand et al., 2012). As a food resource, its meat has a big amount of protein and a reduced percentage of fat content (Avilés et al., 2014). Over the years, the National Institute for Agricultural Innovation (INIA) of Peru developed three breeds of guinea pigs (Andina, Inti, and Peru). These breeds are consumed commonly in the Peruvian gastronomy (Rubio Arias, 2018; Chauca Francia, 2023). Andina breed stands out for its remarkable prolificity (litter size = 3.4), boasting a fertility rate of 98.5% (Rubio Arias, 2018). Inti breed achieves a weight of 0.9 kg at 56 days and a meat carcass yield of 71.1%. Lastly, Peru breed has a great ability to gain weight and meat carcass yield compared to the Andina and Inti breeds (Reynaga Rojas et al., 2020). Peru breed have meat-oriented characteristics, boasting high precocity, fast growth, and prolificity (litter size = 2.8) (Rubio Arias, 2018).

In animal production, the “control” of food intake can influence general aspects of meat and milk composition, quality, and characteristics. Diet alterations in animals will affect muscle composition, fat development, and protein and lipid quality. On the other hand, the health of production animals can be altered by the microbiome of the gastrointestinal tract (GIT), besides is a key component of the development, nutritional absorption, metabolism, and productivity of these animals (Yeoman and White, 2014; Zhang et al., 2021). Therefore, the study of GIT microbiome can generate information and knowledge that allows the improvement of meat. This is one of the reasons for the increasing number of studies about GIT microbiome of production animals, including different guinea pig breeds (Hildebrand et al., 2012; Phillips Campbell et al., 2016; Al et al., 2017; Crowley et al., 2017; Lucking et al., 2018; Palakawong Na Ayudthaya et al., 2019; Shin et al., 2021; Tang et al., 2022; Wada et al., 2022; Frias et al., 2023).

The gut microbiome can be influenced by various factors. For instance, factors such as the host's genetics, diet, fasting, age, and use of antibiotics can all play a role (Francino, 2015; Kurilshikov et al., 2017; Angoorani et al., 2021; Maifeld et al., 2021; Ducarmon et al., 2023; Frias et al., 2023). On the other hand, fasting can impact the gut microbiome in rodents (Angoorani et al., 2021). Additionally, research has shown that host genetics can also influence the effect of fasting on the gut microbiome within the same species (Yan et al., 2021).

The investigation into the effects of fasting on the gut microbiome of guinea pigs remains unexplored thus far. It has been demonstrated by Turley and West (1976) that a 24-h fasting period can lead to a reduction in sterol synthesis in the ileum of guinea pigs at the age of 4 months. Numerous studies have highlighted the potential of sterols in modulating the composition of gut microbiota, resulting in a range of beneficial health effects (Le et al., 2022; Manoppo et al., 2022). Additionally, Langley and Kelly (1992) found that adult guinea pigs, aged 6 months or older, experienced a 9% reduction in body weight following a 48-h fasting period. Weight loss has been found to be linked to alterations in the diversity of the gut microbiota (Jian et al., 2022; Koutoukidis et al., 2022). Based on the aforementioned information, it can be suggested that a 24-h period has the potential to impact the gut microbiome of guinea pigs. The benefits of fasting has been demonstrated in the dietary practices of numerous livestock animal species. The utilization of intermittent fasting as a feeding strategy for chickens is widely acknowledged for its capacity to improve flock uniformity by increasing portion sizes, thereby prolonging feeding periods and reducing feed competition (Lindholm, 2019; Lindholm and Altimiras, 2023). The incorporation of fasting as a pre-slaughter practice for pigs has the potential to enhance carcass hygiene (Faucitano et al., 2010), pork quality, and animal welfare (Driessen et al., 2020). In the context of rabbits, fasting offers several benefits, including enhanced digestive function, a shift in the body's energy allocation from fat to protein, and a decrease in mortality and morbidity associated with digestive issues (Abou-Hashim et al., 2023). Based on the aforementioned information, providing additional information about the impact of fasting on the gut microbiome of guinea pigs can deepen our comprehension and assist in making well-informed decisions regarding the implementation of enhanced feeding strategies that incorporate fasting periods for guinea pigs.

The impact of fasting on the microbiome of livestock animals, specifically guinea pigs, has not been extensively investigated in comparison to studies conducted on humans or mice. The impact of these animals on meat production is currently unknown. Therefore, the present study aims to characterize the microbiome of the cecum in three distinct breeds of guinea pigs (Andina, Inti, and Peru) belonging to two separate groups: a control group (fed group) and a group subjected to a time-restricted fasting regimen (fasting group). The objective is to compare the impact of fasting on the composition of their microbiome. The understanding of these variations and similarities in the circumstances of food limitation can contribute to the advancement of more effective feeding strategies for guinea pigs and enhance the exploration of particular taxa that may be associated with fasting.

## 2 Materials and methods

### 2.1 Ethics statement

The experimental protocol (CIEI-N°005) has received approval from the Institutional Committee on Research Ethics (CIEI) of the National University Toribio Rodriguez de Mendoza (UNTRM).

### 2.2 Criteria for selection, treatment, and sampling of animals

The facilities of the small animal shed of the experimental research center of the Institute of Livestock and Biotechnology (IGBI) of the UNTRM were used for the breeding of the guinea pigs, and the Molecular Physiology Laboratory was used for the molecular procedures.

This study explored whether fasting could modify the population structure of the microbiome of the cecum of three breeds of guinea pigs, To achieve this, we established two treatment groups: fasting and fed, for each of the three guinea pig breeds: Andina, Inti, and Peru. Thirty samples of guinea pig cecum mucosa were used. Still, one of the samples from the Inti breed and one of the samples from the Peru breed did not show enough sequences during the filtering performed and were removed from the analysis, determining 28 samples downstream. The global sample size was 28 male guinea pigs, divided into 10 specimens for the Andina breed (five of the fed group; five of the fasting group), nine specimens for the Inti breed (four of the fed group; five of the fasting group) and nine specimens for the Peru breed (five of the fed group; four of the fasting group). The study involved 70–90-day-old guinea pigs fed with 80% alfalfa and 20% balanced food and a fasting treatment with 24-h fasting before his euthanasia. The bromatological analysis of the alfalfa (*Medicago sativa*) and the balanced food that has been used to feed the guinea pigs was carried out in the Laboratory of Animal Nutrition and Food Bromatology of the UNTRM, where the parameters of humidity (H°), crude protein, ashes, crude fiber, ethereal extract, nitrogen-free extract, according to the protocols established by the same laboratory (Table 1). Guinea pigs were euthanized by cervical dislocation, and microbial samples were obtained from mucosal samples of the cecum.

**Table 1:** Bromatological analysis of alfalfa (*Medicago sativa*) and balanced food.

	**Alfalfa (*Medicago sativa*)**	**Balanced food**
Humidity (H°)	5.14%	12.19%
Crude protein	22.97%	18.24%
Crude fiber	20.59%	10.12%
Ash	9.89%	7.7%
Ethereal extract	2.47%	3.75%
Nitrogen free extract	38.95%	43%

The protocol for collecting samples was modified from Hu et al. (2021). Using sterile swabs, we extracted microbial samples from the cecum. Flushing sterile PBS buffer was used to obtain these samples. After carefully locating the respective body site for each sample, we cut a 1–1.5 cm hole there, collected the contents with sterilized spoons, and then put the contents into sterile microcentrifuge tubes. For each gastrointestinal site (cecum) sample, we used a new, sterilized spoon. We repeatedly gently kneaded 1–2 ml PBS buffer samples for 2 min after flushing them with a sterile syringe. Fifteen milliliter sterile centrifuge tubes were used to collect the lavage fluid, which was then chilled to 4°C. Each sample needed between 5 and 10 ml of lavage fluid, which was then gathered, centrifuged at 4,000 × *g* for 30 min at 4°C to form a pellet, and then transferred into a 2 ml sterile centrifuge tube. The pellet was kept at −80°C until DNA extraction.

### 2.3 DNA extraction, amplification, and sequencing of the 16S rRNA gene

DNA was extracted using the PureLink Genomic DNA Extraction MiniKit (Invitrogen) according to the manufacturer's instructions for Gram-Positive Bacterial Cell Lysate, with a few minor modifications. The “DNA Clean and Concentrator ^®^-5” kit (Zymo Research) was used to purify the extracted genomic DNA. DNA concentration and purity were evaluated using a NanoDrop^®^ Thermo Fisher Scientific Spectrophotometer (Waltham, Massachusetts, USA). Agar gel electrophoresis was used to verify the results.

The DNA samples were sent to the Argonne Laboratory for the amplification and sequencing of the V4 hypervariable region of the bacterial 16S rRNA. The Argonne Laboratory (Argonne, IL, USA) used the MiSeq Reagent Kit V2 with primers 515 F and 806 R created for the Illumina MiSeq platform (Illumina Inc., San Diego, CA) to amplify the V4 hypervariable region of the bacterial 16S rRNA gene from genomic DNA (Caporaso et al., 2011). Degeneracy was added to both the forward and reverse primers to correct known biases against the marine and freshwater *Alphaproteobacterial* clade SAR11 [806R, (Apprill et al., 2015)] and *Crenarchaeota*/*Thaumarchaeota* [515F, also known as 515F-Y, (Parada et al., 2016)].

### 2.4 Sequence and bioinformatics analyses

The Quantitative Insights Into Microbial Ecology 2 (QIIME2) software (v. 2022.11) was used to analyze the microbiome of the cecum of the guinea pigs with the evaluation of the sequences of the V4 hypervariable region of the 16S rRNA gene (Bolyen et al., 2019), on the bioinformatics server of the Molecular Physiology Laboratory of the National University Toribio Rodrguez de Mendoza using Python programming. We used the “DADA2” plugin (v. 1.26.0) (Callahan et al., 2016) to follow the QIIME2 pipeline to execute the demultiplexing of the reads, the trimming process of the sequence adapters, and the deletion of ambiguous, duplicate, low-quality, chimeric, and other sequences. From then, we were only able to continue the analysis up to positions 225 and 193 of the forward and reverse reads, respectively. Additionally, sequences with insufficient amplicon sequence variants (ASVs) per sample were eliminated using alpha rarefaction.

The taxonomic categorization using the SILVA v. 138 database (Quast et al., 2013) and the sklearn classifier was applied to the representative and high-quality sequences to produce the taxonomy tables and ASVs. The data was filtered using the software phyloseq (McMurdie and Holmes, 2013) in R (R Core Team, 2022) to eliminate any ASVs that were unassigned, assigned as being of Archaea, Chloroplast, or Mitochondrial origin, or had no assigned bacterial phylum.

The software R version 4.2.2 (R Core Team, 2022) was used to do all statistical analyses, and a number of packages and techniques were used. Plotting alpha rarefaction curves was done using the vegan package (Oksanen et al., 2022). Utilizing metrics from the indices Shannon diversity (Shannon, 1948), Chao1 richness (Chao, 1984), Abundance-based Coverage Estimator (ACE) of species richness (Chao and Lee, 1992), and Observed operational taxonomic units (OTUs) in the R statistical program, the alpha diversity indices were assessed in the phyloseq package (McMurdie and Holmes, 2013) to compute bacterial diversity. The box plots were created using the MicrobiotaProcess package, and the Alpha diversity box-and-whisker plots were created using the same package (Xu et al., 2023). Analysis of variance (ANOVA; α < 0.05) and the Tukey's honestly-significant-difference (HSD) *post hoc* test were used to compare the index values for the three breed types (Andina, Inti, and Peru) (R Core Team, 2022).

Beta diversity was examined for variations in community structure between different treatments for each breed using the canonical analysis of principal coordinates [CAP; (Anderson and Willis, 2003)] and non-metric multidimensional scaling [NMDS; (44)] methods. These methodologies were used, respectively, in the packages phyloseq (McMurdie and Holmes, 2013) and vegan (Oksanen et al., 2022). A deeper analysis using Permutational Multivariate Analysis of Variance (PERMANOVA) was conducted to assess the differences of the communities among different treatments for each breed with the aid of the function adonis2 from the vegan package (Oksanen et al., 2022) and all the metrics mentioned above over 1,000 permutations. An analysis of similarities (ANOSIM) and an analysis of the multivariate homogeneity of group dispersions were also carried out using the functions anosim and betadisper, respectively. *Post hoc* tests were run in pairs using the pairwise function. Additionally, we used the Euclidean method and Bonferroni correction with the pairwiseAdonis package's adonis to determine the statistical significance of these tests (Martinez Arbizu, 2020).

To compare taxonomic bar plots with relative and absolute abundance at the phylum and genus levels, the microbial composition in the stacked bar plots was analyzed using the R packages qiime2R (Bisanz, 2018) and ggplot2 (Wickham, 2009). The *Firmicutes* to *Bacteroidetes* ratio (F/B ratio) was calculated by dividing the relative abundance of *Firmicutes* by the relative abundance of *Bacteroidetes*, and then the Mann-Whitney U test was used to identify significant statistical differences between the treatment groups for each breed. Through the use of the software microeco (Liu C. et al., 2021), a linear discriminant analysis (LDA) effect size (LEfSe) analysis was conducted to identify the taxa with an LDA of ±2 for effect size among the treatment groups for each breed and their relative abundances. Taxonomic abundance was represented as a differential heat tree using the R package metacoder (Foster et al., 2017), with a Wilcox rank sum test and Benjamin-Hochberg (False discovery rate: FDR) correction for multiple comparisons. Furthermore, lists of the distinct and shared taxa between the treatment groups (for each breed) were created using the packages MicrobiotaProcess (Xu et al., 2023), zoo (Zeileis and Grothendieck, 2005), and VennDiagram (Chen and Boutros, 2011) as well as a Venn diagram showing the different treatments for each breed. The methodology of the present article was based on the methodology described previously in one of our previous articles: Frias et al. (2023).

### 2.5 Data availability

The DNA sequences of the samples used in this investigation can be found in the NCBI SRA repository under the project names BioProject PRJNA956576 (for samples from guinea pigs in the fed groups) and BioProject PRJNA982863 (for samples from guinea pigs in the fasting groups).

## 3 Results

### 3.1 Summary of breeds and sequencing

We used 28 samples of guinea pig cecum's mucosa, 10 samples of the Andina breed (five for the fed group and five for the fasting group), nine samples of the Inti breed (four for the fed group and five for the fasting group), and nine samples of the Peru breed (five for the fed group and four for the fasting group).

A total of 785,617 sequences (Andina), 640,893 sequences (Inti), and 784,554 sequences (Peru) were obtained from sequencing the guinea pig cecum mucosa samples in the V4 region of the 16S rRNA gene. These sequences were used for downstream analyses relevant to the study of the structure and composition of the cecum microbiota of guinea pig breeds treatment groups. On the other hand, the number of reads per sample was: 78,561.700 ± 22,020.000 (mean ± SD) reads/sample (Andina); 71,210.333 ± 25,540.382 (mean ± SD) reads/sample (Inti), and 87,172.667 ± 17,509.425 (mean ± SD) reads/sample (Peru). The median length for all reads was 252.85 bp (Andina), 252.82 bp (Inti), and 252.85 bp (Peru). Overall, 1,716 (Andina), 1,466 (Inti), and 1,680 (Peru) taxa identified were used in the analyses.

### 3.2 Alpha diversity of the cecum microbiota of the guinea pig breeds treatment groups

Rarefaction curves showed that all of the samples had reached the saturation plateau, demonstrating that the volume of sequencing data was sufficient and could accurately represent the vast majority of the microorganisms in the samples (Supplementary Figure S1 in Supplementary Presentation 1). To determine if there were any differences among the sample groups, richness (Chao1 index) and diversity (Shannon index) were examined. The Shannon and Chao1 indices did not differ statistically by treatment group within samples (Tables S1–S9 in Supplementary Data Sheet 1). There was a tendency for a higher Chao1 index and the number of Observed OTUs of the fed group in comparison to the fasting group in the Inti and Peru breeds, but not in the Andina breed. On the other hand, there was a tendency for a higher Shannon index of the fasting group in comparison to the fed group in the Inti and Peru breeds, but not in the Andina breed.

**Figure 1:**
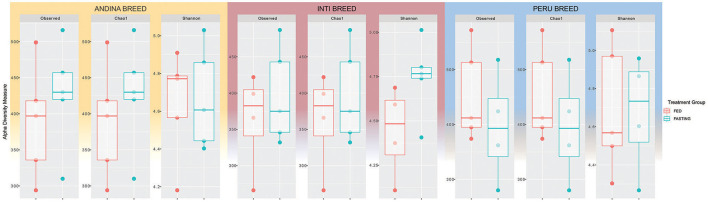
Box plots representing alpha diversity indices for the comparison of guinea pig breeds treatment groups. Different colors indicate different treatments (red: fed and green: fasting) for each breed: Andina **(left)**, Inti **(center)**, and Peru **(right)**. The horizontal line inside the boxes represents the median, the box indicates the interquartile range, and the thin vertical black line represents the rest of the distribution.

Interestingly, we observed in the figures that there were slight changes in the structure of alpha diversity among samples of all breeds. The Andina breed increased the richness index (Chao1) and decreased sample diversity (Shannon). On the other hand, the Inti and Peru breeds had a slight decrease in richness and an increase in diversity. This demonstrates that statistically fasting does not change alpha diversity, but the graphical data show a slight change (Figure 1).

### 3.3 Differences in microbial composition among groups based on beta diversity

**Figure 2:**
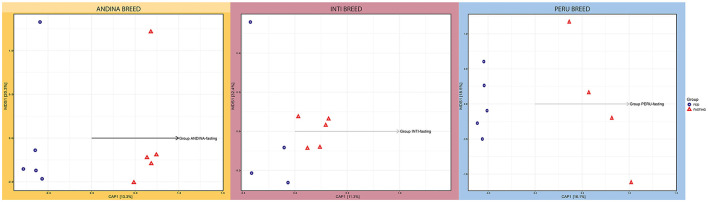
Canonical Principal Coordinate Analysis (CAP) of the guinea pig breeds treatment groups. The forms indicate the types of treatment: a triangle for the fasting group and a circle for the fed group in each breed: Andina **(left)**, Inti **(center)**, and Peru **(right)**. The arrangement of the arrows illustrates the formation of groups of individuals selected in different coordinates, indicating the dissimilarity and similarity of microbiota composition among the independent samples and groups of treatments within breed groups. The CAP was built on an unweighted UniFrac distance. CAP, Canonical Principal Analysis; NMDS, Non-metric MultiDimensional Scaling.

CAP and NMDS were used to investigate the beta diversity of microbial communities. The CAP in Figure 2 illustrates how the treatment groups differ from one another in each breed (Andina, Inti, and Peru). A clear separation of the treatment groups was found in the Andina and Peru breeds, but a slight separation for Inti.

However, we only found a significant difference between the treatment groups of the Peru breed (*P*-value < 0.05), but not for the Andina and Inti breeds (Supplementary Tables S10–S12 in Supplementary Data Sheet 1). Following qualitative ecological data of the various bacterial species grouped, the intestinal microbiota of the Peru breed varies between the treatment groups. The treatment groups of each breed were separated based on unique fraction metric (Unifrac) unweighted distances (which take into account the presence or absence of a species) and Unifrac weighted distances (which take into account information about species abundance), as shown in the Supplementary Figures S2, S3. These distances are based on phylogenetic distance measurements and were used in the NMDS plots. The NMDS plots showed no evident clustering of treatment groups for each breed. Based on unweighted Unifrac distances, only the treatment groups of the Peru breed displayed significant similarity, with an even distribution of high and low ranks within and between groups, in the NMDS plot (ANOSIM: *R*^2^ = 0.3563; *P-*value = 0.04).

### 3.4 Differences in the composition of the cecum microbiota of the guinea pig breeds treatment groups

**Figure 3:**
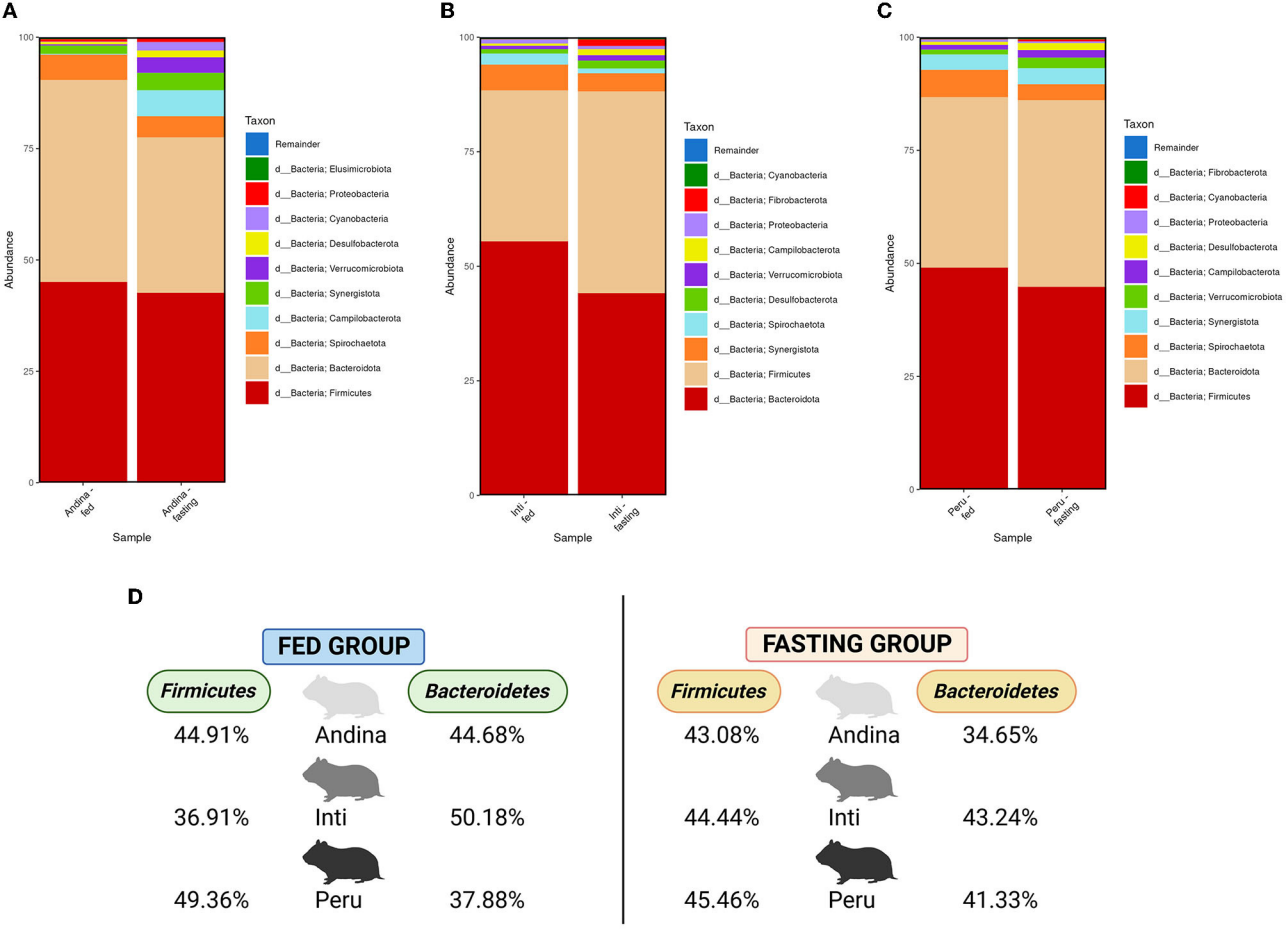
Relative abundance of the main phyla in the guinea pig breeds treatment groups. The 10 main phyla of the cecum microbiota present in the treatment groups (fed and fasting groups) within guinea pig breed groups: Andina **(A)**, Inti **(B)**, and Peru **(C)**. Representation of the relative abundance percentages of the two main phyla present in all the treatment breed groups **(D)**. d, domain. The subfigure **(D)** was created with BioRender.com.

**Figure 4:**
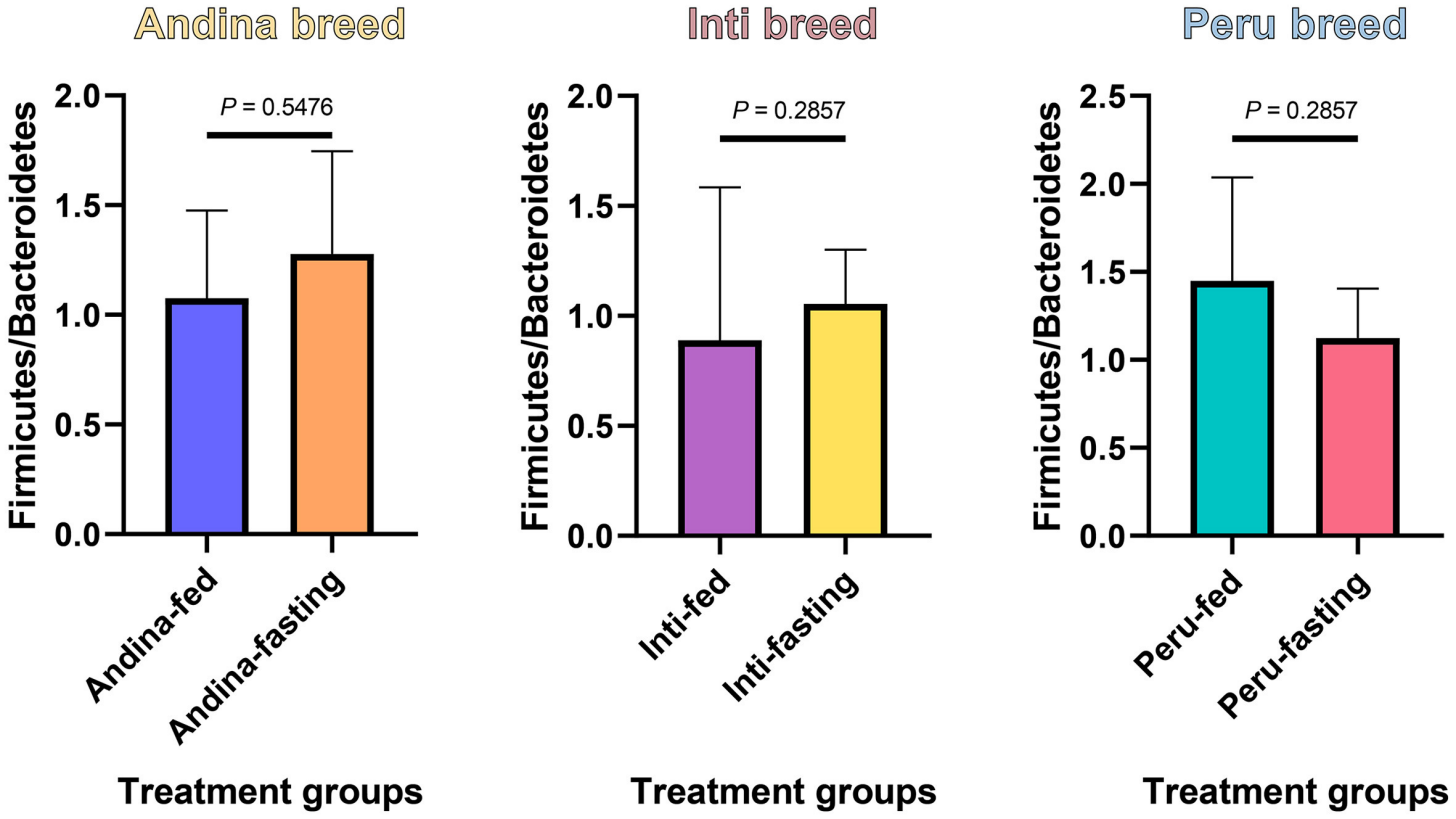
The ratio of Firmicutes to Bacteroidetes in the guinea pig breed treatment groups. The *P*-values were obtained from the Mann–Whitney *U*-test. The box and whisker represent the mean ± standard deviation (SD).

**Figure 5:**
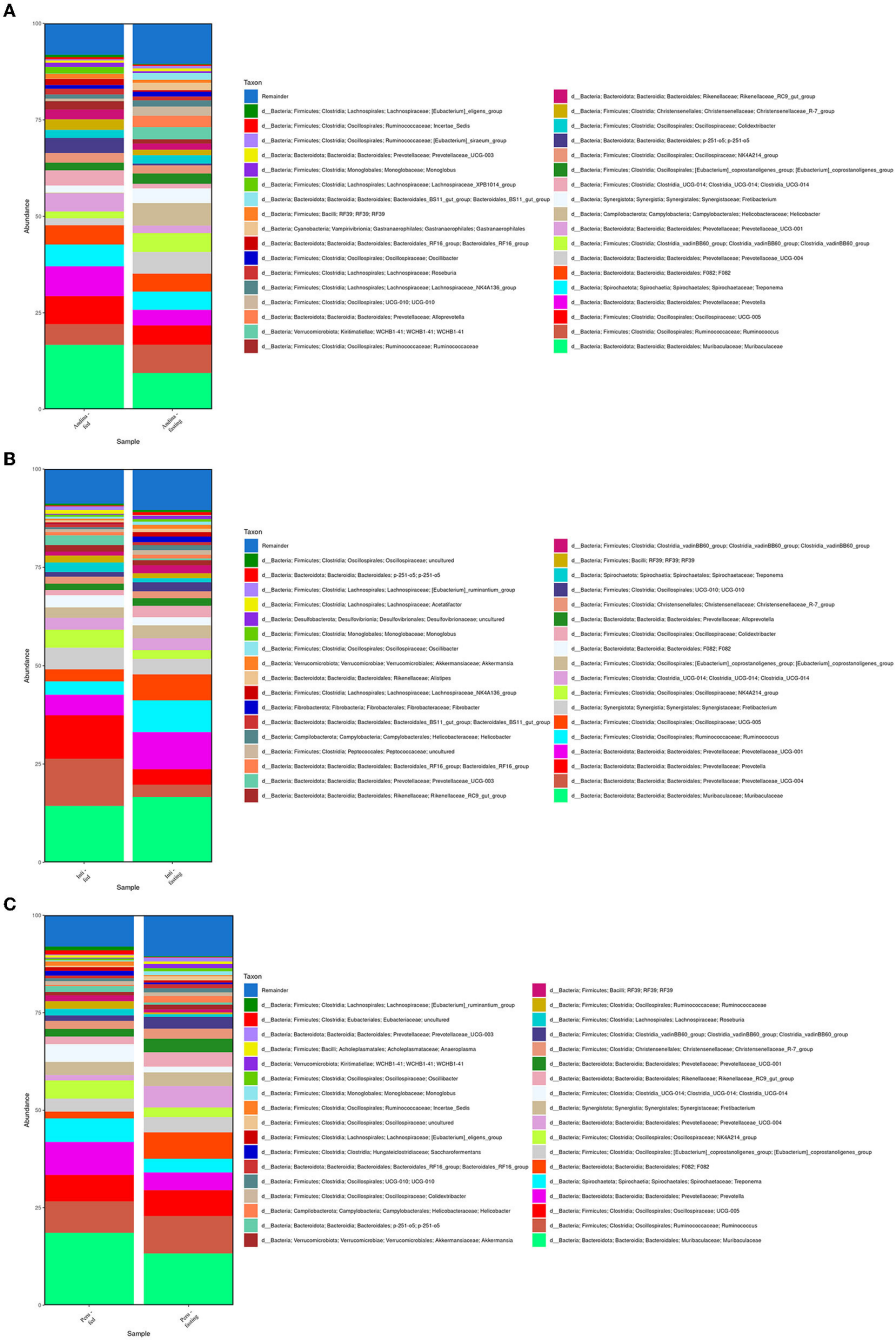
Relative abundance of the 35 main genera present in the guinea pig treatment breed groups. The relative abundance of the 35 main genera present in the cecum microbiota of the treatment groups (fed and fasting groups) within guinea pig breed groups: Andina **(A)**, Inti **(B)**, and Peru **(C)**. d, domain.

**Figure 6:**
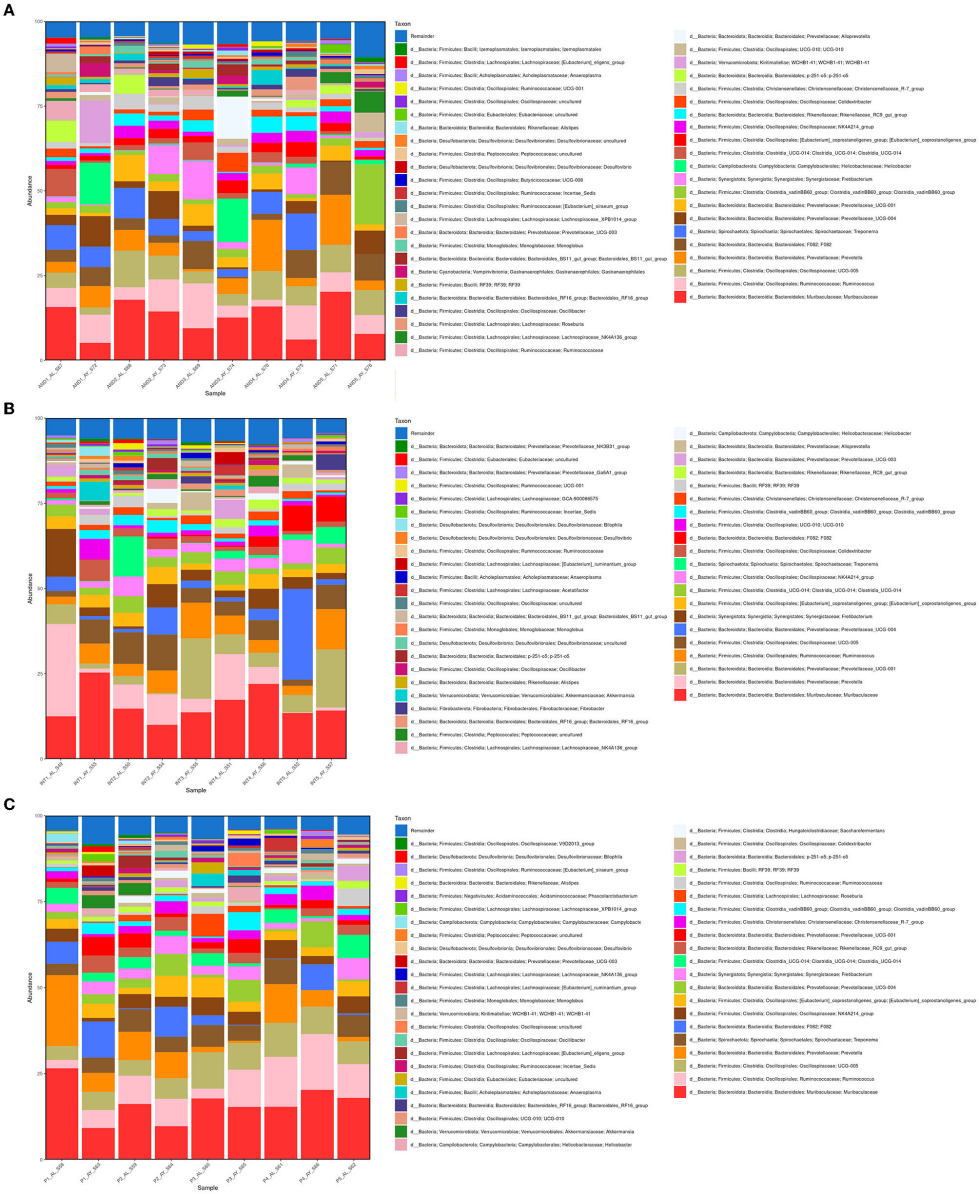
Relative abundance of the 45 main genera of the cecum microbiota present in the samples. The relative abundance of the 45 main genera present in the cecum microbiota of the treatment groups (fed and fasting groups) within guinea pig breed groups: Andina **(A)**, Inti **(B)**, and Peru **(C)**. d, domain; INT, Inti breed; AND, Andina breed; P, Peru breed; AL, fed treatment; AY, fasting treatment.

The microbial compositions present in the cecum microbiota of guinea pigs from different treatment groups within breeds are shown in Figures 3–**6**. The figures of the absolute microbiota composition are displayed in Supplementary Figures S4–S6.

In Figure 3, among the 10 main phyla that we found in the cecum microbiota of the guinea pig breeds treatment groups, we found that *Firmicutes* and *Bacteroidetes* were the 2 most abundant phyla for all the guinea pig breeds treatment groups.

In Figure 4, we found an increase in the F/B ratio in the fasting groups of the Andina and Inti breeds but not for the Peru breed, in this case, we found an opposite effect. Denote, that the animals were maintained in the same environment and received the same food/fasting procedures.

We found that the most dominant genus in all groups was *Muribaculaceae* (*Muribaculaceae*), with variable rates of 15.85% and 9.24% for the Andina-fed and Andina-fasting groups; 14.53% and 17.06% for Inti-fed and Inti-fasting groups; 18.78% and 13.65% for the Peru-fed and Peru-fasting groups, respectively (Figure 5).

Also, in the majority of the samples of all groups, the most dominant genus was *Muribaculaceae* (*Muribaculaceae*; Figure 6). The other two predominant genera after *Muribaculaceae* were *Prevotella* (7.99%) and *Oscillospiraceae* (UCG-005) for the Andina-fed group, *Ruminococcus* (7.36%) and *Prevotellaceae* (*Prevotellaceae* UCG-004; 5.69%) for the Andina-fasting group, *Prevotella* (11.93%) and *Prevotellaceae* (*Prevotellaceae* UCG-004; 8.26%) for the Inti-fed group, *Prevotellaceae* (*Prevotellaceae* UCG-001; 8.17%) and *Ruminococcus* (7.70%) for the Inti-fasting, *Prevotella* (8.62%) and *Ruminococcus* (7.55%) for the Peru-fed group, *Ruminococcus* (10.03%) and *Oscillospiraceae* (UCG-005; 6.75%) for the Peru-fasting group. The values of the whole taxa relative abundance have been stored in the Supplementary Data Sheet 2.

**Figure 7:**
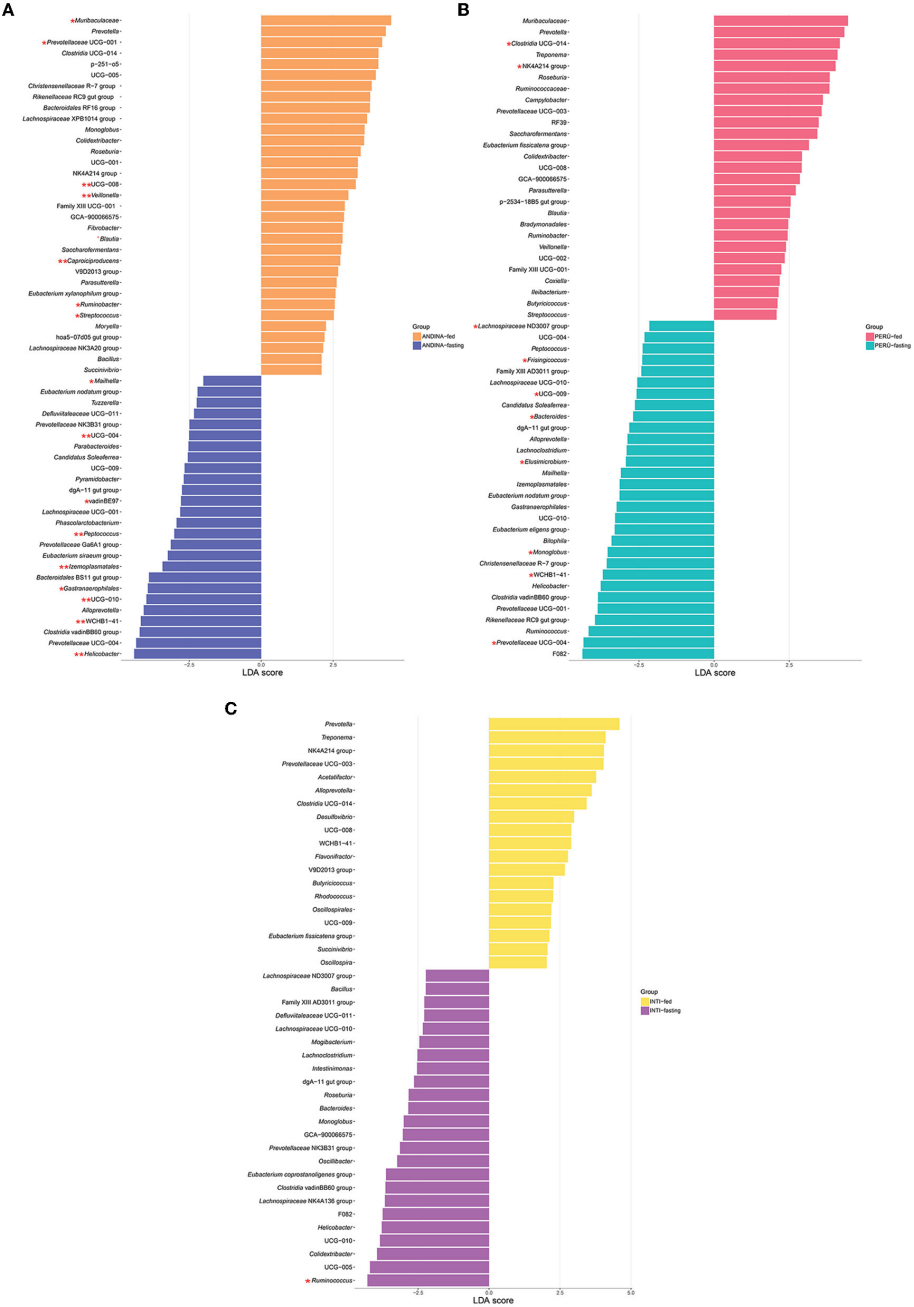
Linear discriminant analysis (LDA) effect size (LEfSe) comparison of the guinea pig breeds treatment groups. LefSe comparison of differentially abundant bacterial taxa between the treatment groups (fed and fasting groups) within the breeds of the guinea pig: Andina **(A)**, Peru **(B)**, and Inti **(C)**. Horizontal bars represent the effect size for each taxon. LDA score cutoff of 2.0 was used to determine an enrichment in a bacterial taxon. One red asterisk (*P* < 0.05) and two red asterisks (*P* < 0.01) denote taxa with statistically significant differences between the abundances of treatment groups within breed groups.

We identified an enrichment of 59 taxa in the Andina breed, 43 taxa in the Inti breed, and 57 taxa in the Peru breed (Figure 7). Four taxa were enriched in all the fasting groups: *Clostridia* (genus *Clostridia* vadinBB60 group), *Rikenellaceae* (dgA-11 gut group), *Helicobacter*, and *Oscillospirales* (genus UCG-010). Also, we found 28 taxa that have a significant enrichment (*P* < 0.05; LDA score >2; LDA score < -2) with the LefSe comparison in some of the treatment groups. For the Andina-fed group, the significantly enriched taxa were *Muribaculaceae* (*Muribaculaceae*), *Prevotellaceae* (*Prevotellaceae* UCG-001), *Blautia, Ruminobacter, Streptococcus, Butyricicoccaceae* (UCG-008), *Veillonella*, and *Caproiciproducens*. For the Andina-fasting group, the significantly enriched taxa were *Gastranaerophilales* (genus *Gastranaerophilales*), *Victivallales* (genus vadinBE97), *Helicobacter, Kiritimatiellae* (genus WCHB1-41), *Oscillospirales* (genus UCG-010), I*zemoplasmatales* (genus *Izemoplasmatales*), *Peptococcus*, and *Erysipelatoclostridiaceae* (UCG-004) were the enriched taxa. For the Inti-fasting group, the significantly enriched taxa were *Ruminococcus*. For the Peru-fed group, the significantly enriched taxa were *Clostridia* (genus *Clostridia* UCG-014) and *Oscillospiraceae* (NK4A214 group). For the Peru-fasting group, the significantly enriched taxa were *Prevotellaceae* (*Prevotellaceae* UCG-004), *Kiritimatiellae* (genus WCHB1-41), *Monoglobus, Elusimicrobium, Bacteroides, Butyricicoccaceae* (UCG-009), *Frisingicoccus*, and *Victivallales* (genus vadinBE97). The complete data of the LefSe comparison has been stored in the Supplementary Data Sheet 3.

**Figure 8:**
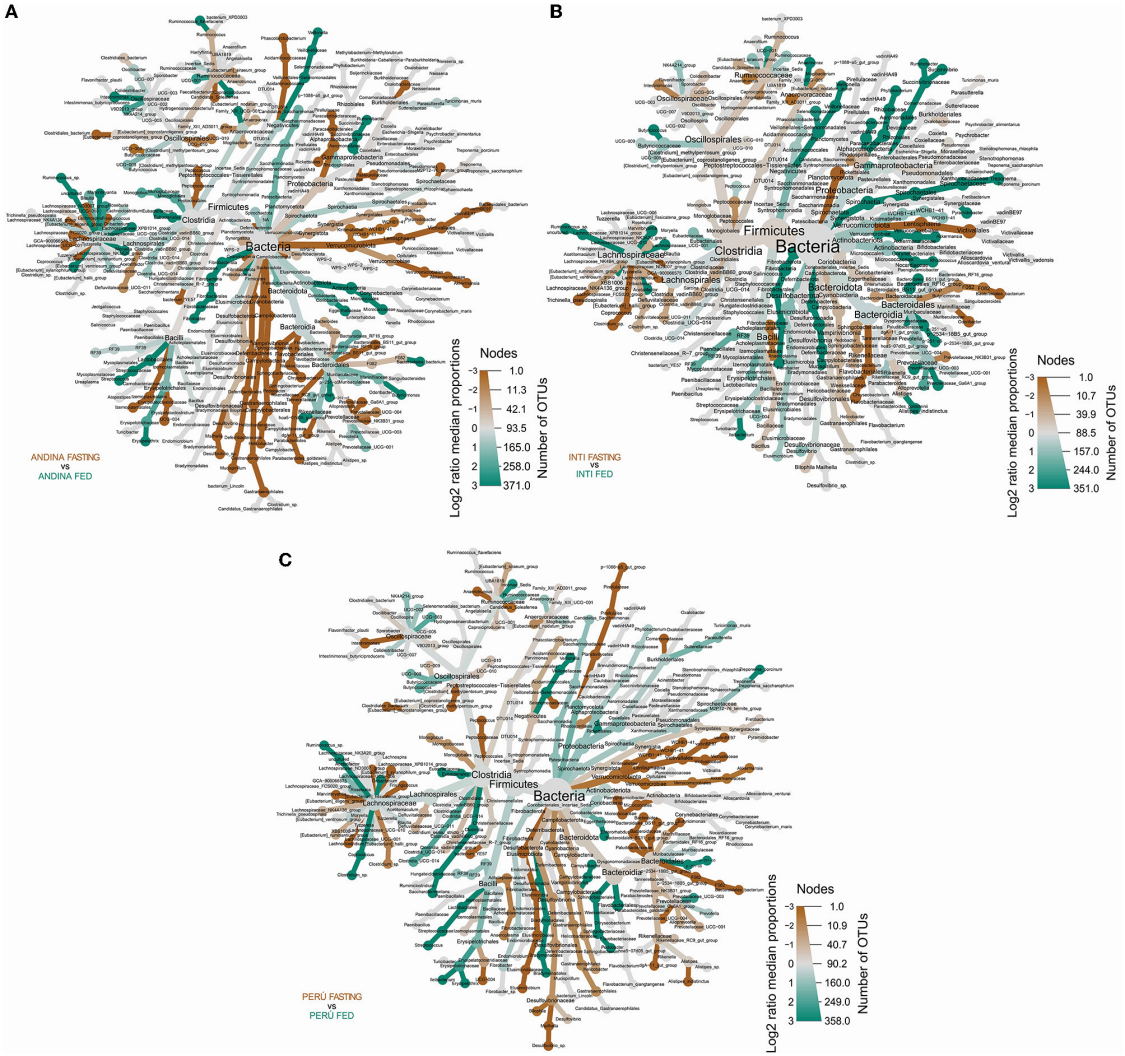
Differential heat tree comparison between the guinea pig breeds treatment groups. The differential heat trees show the taxonomy and the comparison between the taxa of the treatment groups (fed and fasting groups) within the breed groups: Andina **(A)**, Inti **(B)**, and Peru **(C)**. The color and the size of the nodes and edges correlate with the richness or number of operational taxonomic units (OTUs) of organisms within the community in which they are found. Color intensity is related to the log_2_ ratio of the difference in median proportions and the Wilcoxon test applied to the readings in each treatment group (fed and fasting groups) for each breed group. The brown taxa show an enhancement in the fasting group, and the green taxa show the opposite in the other comparative group (fed group) within the breed groups: Andina **(A)**, Inti **(B)**, and Peru **(C)**. In gray, the nodes are equally present in both compartments. OTUs, operational taxonomic units.

The heat tree analysis showed 45 taxa with a significant difference between the treatment groups in the Andina breed, six taxa between the treatment groups of the Inti breed, 23 taxa between the treatment groups of the Peru breed (Figure 8, Supplementary Data Sheet 4).

We found several differences between the treatment groups in the Andina breed: in the phylum level: *Campylobacterota*: *Campylobacteria* (*Campylobacterales*); *Cyanobacteria*: *Vampirivibrionia* [*Gastranaerophilales* (family and genus *Gastranaerophilales*)]; and *Verrumicrobiota*: *Kiritimatiellae* (order, family, and genus WCHB1-41) and *Lentisphaeria* [*Victivallales* (family and genus vadinBE97)]. In the order level: *Izemoplasmatales* (family and genus *Izemoplasmatales*); *Oscillospirales* [family and genus UCG-010, *Butyricicoccaceae* (UCG-008)]; *Peptococcales*: *Peptococcaceae* (*Peptococcus*); and *Eubacteriales* (*Eubacteriaceae*). In the family level: *Veillonellaceae* (*Veillonella*); *Helicobacteraceae* (*Helicobacter*); *Erysipelatoclostridiaceae* (UCG-004); *Streptococcaceae* (*Streptococcus*); *Muribaculaceae* (*Muribaculaceae*); and *Prevotellaceae* (*Prevotellaceae* UCG-001). In the genus level: *Desulfovibrio, Mailhella, Ruminococcus, Caproiciproducens*, and *Blautia*.

We found several differences between the treatment groups in the Inti breed: in the phylum level: *Spirochaetota* (*Spirochaetia*). In the family level: *Weeksellaceae* (*Chryseobacterium*). In the genus level: *Ruminococcus*. In the species level: *Trichinella pseudospiralis*.

We found several differences between the treatment groups in the Peru breed: in the class level: *Clostridia* (order, family, and genus *Clostridia* UCG-014) and *Kiritimatiellae* (order, family, and genus WCHB1-41). In the order level: *Eubacteriales* (*Eubacteriaceae*); *Peptococcales* (*Peptococcaceae*); *Monoglobales*: *Monoglobaceae* (*Monoglobus*). In the family level: *Butyricicoccaceae* (UCG-009); *Ruminococcaceae* (*Incertae Sedis*); *Oscillospiraceae* (NK4A214 group); *Lachnospiraceae* (*Lachnospiraceae* ND3007 group and *Frisingicoccus*); *Bacteroidaceae* (*Bacteroides*); and *Prevotellaceae* (*Prevotellaceae* UCG-004).

**Figure 9:**
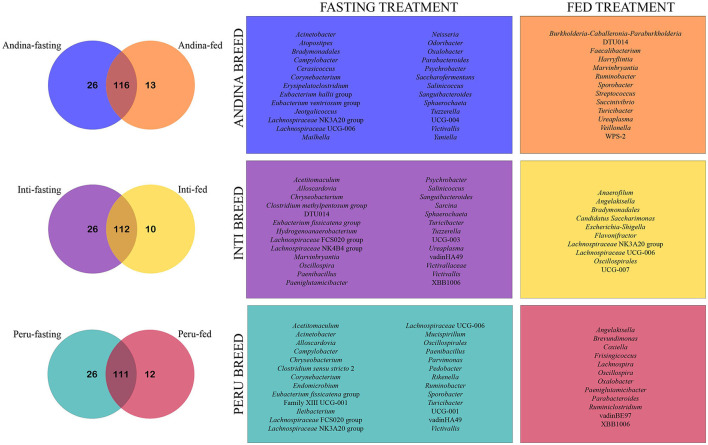
Venn chart illustrating unique and shared genera among different guinea pig breeds treatment groups. The Venn charts illustrate the comparison of the unique and shared genera present in the treatment groups (fed and fasting) for each breed group (Andina, Inti, and Peru). The numbers inside the subsets on the Venn diagrams represent the number of genera identified for each subset (unique breed-fasting subset, unique breed-fed subset, and shared subset). On the right side of the Venn diagrams, the frames are labeled with the colors of their respective subsets and show each unique genus that has been cataloged for their subsets. Some genera may have the same name but different amplicon sequence variants (ASVs).

The Venn diagram shows that most of the taxa were shared between the treatment groups within the breed groups (Andina: 116 taxa, Inti: 112 taxa, and Peru: 111 taxa). Furthermore, we found a greater number of unique taxa in the fasting groups in comparison with the fed groups of the different breeds. Also, the only unique genus that the fasting groups of the three breeds have in common is *Victivallis* (Figure 9). The shared taxa between treatment groups of each breed group were specified in the Supplementary Data Sheet 5.

## 4 Discussion

The cecum microbiota of the guinea pig plays a key role in the fermentation of the vegetal material eaten by the animal (Tang et al., 2022; Frias et al., 2023). To the best of our knowledge, this is the first study that explores the effect of fasting on the cecum microbiome of the guinea pig Andina, Inti, and Peru breeds. The alpha diversity between the samples of the fasting and fed groups showed an observational difference between the samples and beta diversity demonstrated that the Peru breed may be the most affected by the fasting period. For the composition of the microbiota, we identified notable changes or fluctuations in the taxonomy, with the *Firmicutes* to *Bacteroidetes* relationship possibly being the most affected. However, the family *Muribaculaceae*, being the most abundant, was the most present among the breeds and the most resilient to the fasting period. We also found a higher number of unique genera in the fasting groups of the breeds.

In the analysis of the alpha diversity, we found that there was an increase in the richness for the fasting group in the Andina and Inti breeds, but not in the Peru breed. Also, there was an increase in the diversity for the fasting group in the Inti and Peru breeds, but not for the Andina breed. Some studies have found that intermittent fasting can increase the gut microbiome richness (Observed OTUs) and the gut microbiome diversity (Simpson index) in a diabetic model and diet-induced obese mice, respectively (Deng et al., 2020; Liu et al., 2020). In the analysis of the beta diversity, we found a significant difference between the bacterial communities of the treatment groups of the Peru breed, but not for the other breeds. Based on these findings we could argue that the fasting treatment can have a significant effect on the bacterial communities of the cecum microbiota of the Peru breed, but not in the other breeds. These findings are related to other studies in humans and mice: Ali et al. (2021) identified that fasting can significantly change the beta diversity in the gut microbiome of humans. Also, Daas and de Roos (2021) report that multiple studies that investigated the effects of fasting on the gut microbiome of mice have similar results regarding the increase of beta diversity.

There are several phenotypic differences between the guinea pig breeds: Andina, Inti, and Peru. For example, the Peru breed has a major meat carcass yield and weight gain in comparison with Andina and Inti, when fed with the same feeding system (Reynaga Rojas et al., 2020). These phenotypic differences between the Peru breed with the Andina and Inti breed could be associated with the compositional differences in the microbiota of the cecum that the Peru breed presents in his fasting state and fed state, as detailed in the present study.

The phyla that dominated all guinea pig breeds treatment groups were *Firmicutes* and *Bacteroidetes*, normally found in the cecum microbiota of guinea pigs (Tang et al., 2022; Frias et al., 2023), and several studies detailed their importance for fatty acid production through the fermentation of different substrates (Thomas et al., 2011; Rowland et al., 2018; Parada Venegas et al., 2019; Stojanov et al., 2020). These two phyla are important for fatty acid production through fermentation. However, these phyla are the ones that may have fluctuated the most during the fasting period, this was assessed in this study with the analysis of the F/B ratio. The F/B ratio is associated with intestinal homeostasis. The balance of the intestinal ecosystem is critical for maintaining normal body function and significant changes in the ratio F/B can be meaningful to the health aspect of the organism (Stojanov et al., 2020). The F/B ratio was calculated by dividing the relative abundances of *Firmicutes* by the relative abundance of the *Bacteroidetes*, like in previous studies (Houtman et al., 2022; Tang et al., 2022). We found a reduction of the F/B ratio in the fasting group of the Peru breed, and in the long term, this could influence the transformation of nutrients into the necessary fat (Deng et al., 2020; Angoorani et al., 2021). Based on the aforementioned, we could suggest that the Peru breed has a higher susceptibility to the fasting treatment.

Furthermore, we observed an increase in the relative abundance of the genera *Akkermansia* and *Bacteroides* in the fasting groups of all three breeds. Members of the genus *Akkermansia* are mucolytic bacteria and do mucin forage and use host-derived substances, similar to members of *Bacteroides* that have a growth advantage over the other populations of bacteria that rely strictly on dietary substrates (Sonoyama et al., 2009; Ducarmon et al., 2023). Furthermore, we also observed an increase in the relative abundance of the genera from the family *Desulfovibrionaceae*. The majority of the members of this family are sulfate-reducing bacteria (Spring et al., 2019), and the increase in this family could be related to the fact that the increased mucin foraging during the fasting treatment can cause the proliferation of sulfur-reducing bacteria (Ducarmon et al., 2023).

The LefSe analysis contributed to the identification of four taxa that were enriched in all the fasting groups of the three breeds: *Clostridia* (genus *Clostridia* vadinBB60 group), *Rikenellaceae* (dgA-11 gut group), *Helicobacter*, and *Oscillospirales* (genus UCG-010). Furthermore, Maifeld et al. (2021) found that members of *Clostridia* showed an opposite effect with the fasting treatment in the gut microbiome of humans. An enrichment in the family *Rikenellaceae* was already found in the gut microbiome of rodents after a fasting treatment: Zhang et al. (2020) determined an increase of *Rikenellaceae* in the intermittent energy fasting group in the gut microbiome of a colitis mouse model and Su et al. (2022) found similar results in the gut microbiome of BALB/c mice after Ramadan fasting. We could argue that the increase in the abundance of the members of *Helicobacter* in the fasting groups could be related to a reduction in the microbiome-mediated colonization resistance against potential pathogens of the gut microbiota of the guinea pig (Ducarmon et al., 2023). On the other hand, Liu J. et al. (2021) found that an intermittent fasting treatment in mice can cause a significant reduction in the abundance of *Helicobacter*. Some members of the order *Oscillospirales* can metabolize sugars and produce short-chain fatty acids (SCFAs) as fermentation products (Yang et al., 2021).

We found several abundance differences between the treatment groups in different taxonomic ranks with the Heat tree analysis. The fasting treatment significantly increased the abundance of *Bacteroidetes, Bacteroidia, Lachnospirales*, and *Lachnospiraceae* in the Inti and Peru breeds, but not in the Andina breed, in this breed decreased. Mesnage et al. (2019) found that the Buchinger fasting treatment led to an increase in the abundance of *Bacteroidetes*, which uses derived energy substrates. Also, Su et al. (2021) discovered that the Ramadan fasting treatment increased the amount of *Lachnospiraceae*, a family of bacteria that break down dietary polysaccharides (Angoorani et al., 2021).

The fasting treatment significantly increased the abundance of *Firmicutes, Clostridia*, and *Oscillospirales* in the Inti breed, but not in the Andina and Peru breeds, in these breeds, some inferior taxonomic ranks showed an increased or decreased abundance. Angoorani et al. (2021) reported that the Ramadan fasting treatment led to a decreasing trend in the abundance of *Firmicutes*. Sonoyama et al. (2009) found that the fed active group led to an increase in the abundance of *Clostridia* (compared with a fasting active group, which fasted for 96 hours) in the cecum microbiome of hamsters. Kohl et al. (2014) found that the fasting treatment led to an increase in the abundance of *Oscillospirales* in the cecum microbiome of mice, tilapia, and quail.

The fasting treatment significantly increased the abundance of *Ruminococcaceae* in the Inti breed, but not in the Andina and Peru breeds, in these breeds decreased. Mesnage et al. (2019) found that a Buchinger fasting treatment reduced the abundance of *Ruminococcaceae*. On the other hand, Su et al. (2021) found the opposite effect with a Ramadan fasting treatment.

There are several reports that host genetics can influence the differences in the abundance of the microorganisms present in the gut microbiota of guinea pig breeds (Frias et al., 2023), pig breeds (Bergamaschi et al., 2020), chicken breeds (Sun et al., 2018; Yan et al., 2021), dog breeds (Morelli et al., 2022), and mice breeds (Campbell et al., 2012). These differences then could have an impact on the effect of a fasting treatment or a feed restriction treatment such as that observed in the cecal microbiome of different chicken breeds (Yan et al., 2021). Furthermore, fasting has been shown to elicit weight loss and inhibit sterol synthesis in the ileum of guinea pigs (Turley and West, 1976; Langley and Kelly, 1992). These two effects have also been observed in conjunction with alterations in the gut microbiome of other animal species (Jian et al., 2022; Koutoukidis et al., 2022; Le et al., 2022; Manoppo et al., 2022). Hence, based on the aforementioned evidence, it can be argued that the differences in the impact of fasting among guinea pig breeds at different taxonomic levels may be attributed to a potential genetic influence of the host on the cecum microbiota.

Finally, we found unique genera in all the treatment groups within the three breeds of guinea pigs. The fasting group has more unique genera than the fed group in all three breeds, this could be related to the finding made by another study that identified an increase in the richness of the fasting group in the gut microbiome of humans (Ozkul et al., 2020). The only unique genus that the fasting groups of the three breeds shared was *Victivallis*. This genus has members that are cellobiose-degrading, produce SCFAs, and are positively associated with fat-derived energy from dietary intake in humans (Zoetendal et al., 2003; Ali et al., 2021).

As we explained, fasting seems to direct to a more homogeneous group of microbes. However, there are lots of different strategies for fasting that could lead to different changes in the composition of the microbiome, for example, the most representative are Buchinger fasting (calorie-restricted regimen) and Ramadan fasting (time-restricted regimen) that have some different effects in the gut microbiota of several animals and humans (Angoorani et al., 2021). However, there are no reports of the effect of fasting on the cecum microbiota of guinea pigs. Therefore, the interaction between fasting and the cecum microbiome from guinea pigs can be further clarified by our research. But understanding more of the effects of prolonged fasting or intermittent fasting should be applied, expecting a long-term reflection of the microbiome and a more complex understanding. Intermittent fasting promotes microbial fermentation, forming several bioproducts with beneficial effects on metabolic disorders (Li et al., 2017). This could be one future approach led by this research since the microbiome, as it is known, can influence several biological aspects in humans and animals. Additionally, future research endeavors may contemplate the inclusion of extended fasting durations preceding euthanasia or delve into the examination of feeding strategies that encompass intermittent fasting.

## 5 Conclusions

Ceasing food intake can modify the structure of the microbiome. The current study discovered a different effect of fasting on the cecum microbiome of the guinea pigs: Andina, Inti, and Peru breeds. The analysis of the beta diversity shows significant differences only between the treatment groups of the breed Peru, but not for the other breeds. We found that two main phyla were shared between all the guinea pig breeds treatment groups: *Bacteroidetes* and *Firmicutes*, with fluctuations in the bacterial count after fasting. Additionally, we discovered that the Peru breed was the only breed that demonstrated that the fasting treatment reduced the F/B ratio. Although we found unique genera in all the guinea pig breeds treatment groups, the only unique genus that the fasting groups of the three breeds shared was *Victivallis*. Finally, this study is the first to elucidate how fasting can affect the cecum microbiome of different breeds of guinea pigs. Despite the results found, the resilience of the gut microbiome was not challenged, causing disruptive changes that can influence the general maintenance of the cecum microbiome. Although, based on the several differences found in the treatment groups of the Peru breed, we could suggest that the fasting treatment has a bigger effect on this breed.

## Data availability statement

The datasets presented in this study can be found in online repositories. The names of the repository/repositories and accession number(s) can be found at: https://www.ncbi.nlm.nih.gov/bioproject/PRJNA956576, PRJNA956576; https://dataview.ncbi.nlm.nih.gov/object/PRJNA982863?reviewer=il3qiric5go0ur1n6uln77u5jv, PRJNA982863.

## Ethics statement

The experimental protocol (CIEI-N°005) has received approval from the Institutional Committee on Research Ethics (CIEI) of the National University Toribio Rodriguez de Mendoza (UNTRM). The study was conducted in accordance with the local legislation and institutional requirements.

## Author contributions

HF: Conceptualization, Investigation, Writing – original draft, Writing – review & editing. NM: Conceptualization, Investigation, Writing – original draft, Writing – review & editing. GF: Conceptualization, Investigation, Writing – original draft, Writing – review & editing. VC: Data curation, Formal Analysis, Methodology, Software, Writing – original draft. AR: Investigation, Writing – review & editing. WB: Writing – review & editing. GS: Writing – review & editing. RP: Investigation, Methodology, Writing – review & editing. DV: Investigation, Writing – original draft. ER: Writing – review & editing. RL: Project administration, Supervision, Writing – review & editing. JM: Project administration, Supervision, Writing – review & editing.
