# Supplementary material for: Comparative analysis of fasting effects on the cecum microbiome in three guinea pig breeds: Andina, Inti, and Peru
Source: Front Microbiol. 2023 Dec 20;14:1283738. doi: 10.3389/fmicb.2023.1283738 (PMC10761435; doi:10.3389/fmicb.2023.1283738)
Supplement: Supplementary file 1 [file Presentation_1.PPTX]

## Slide 1
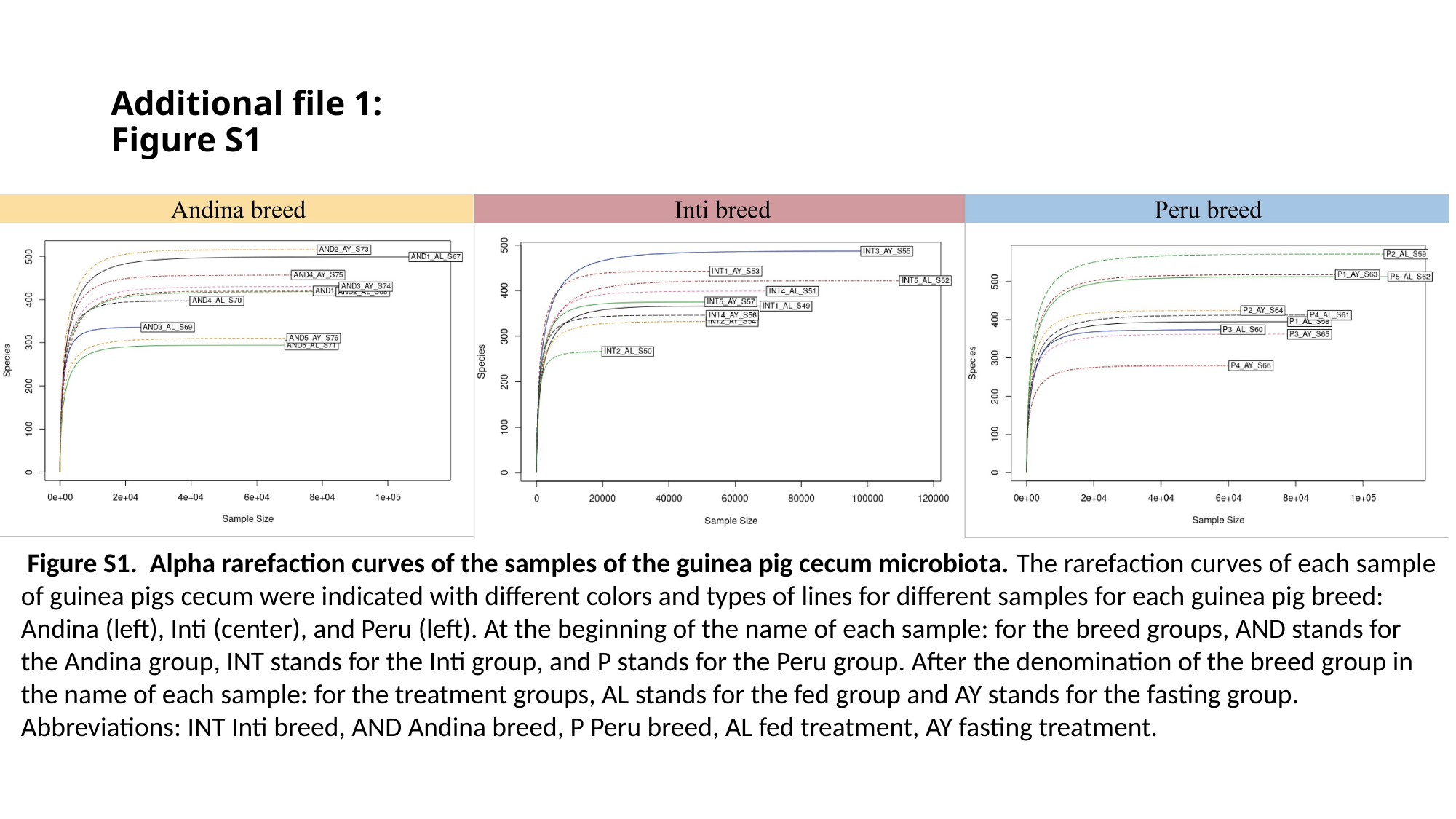

# Additional file 1: Figure S1
 Figure S1. Alpha rarefaction curves of the samples of the guinea pig cecum microbiota. The rarefaction curves of each sample of guinea pigs cecum were indicated with different colors and types of lines for different samples for each guinea pig breed: Andina (left), Inti (center), and Peru (left). At the beginning of the name of each sample: for the breed groups, AND stands for the Andina group, INT stands for the Inti group, and P stands for the Peru group. After the denomination of the breed group in the name of each sample: for the treatment groups, AL stands for the fed group and AY stands for the fasting group. Abbreviations: INT Inti breed, AND Andina breed, P Peru breed, AL fed treatment, AY fasting treatment.

## Slide 2
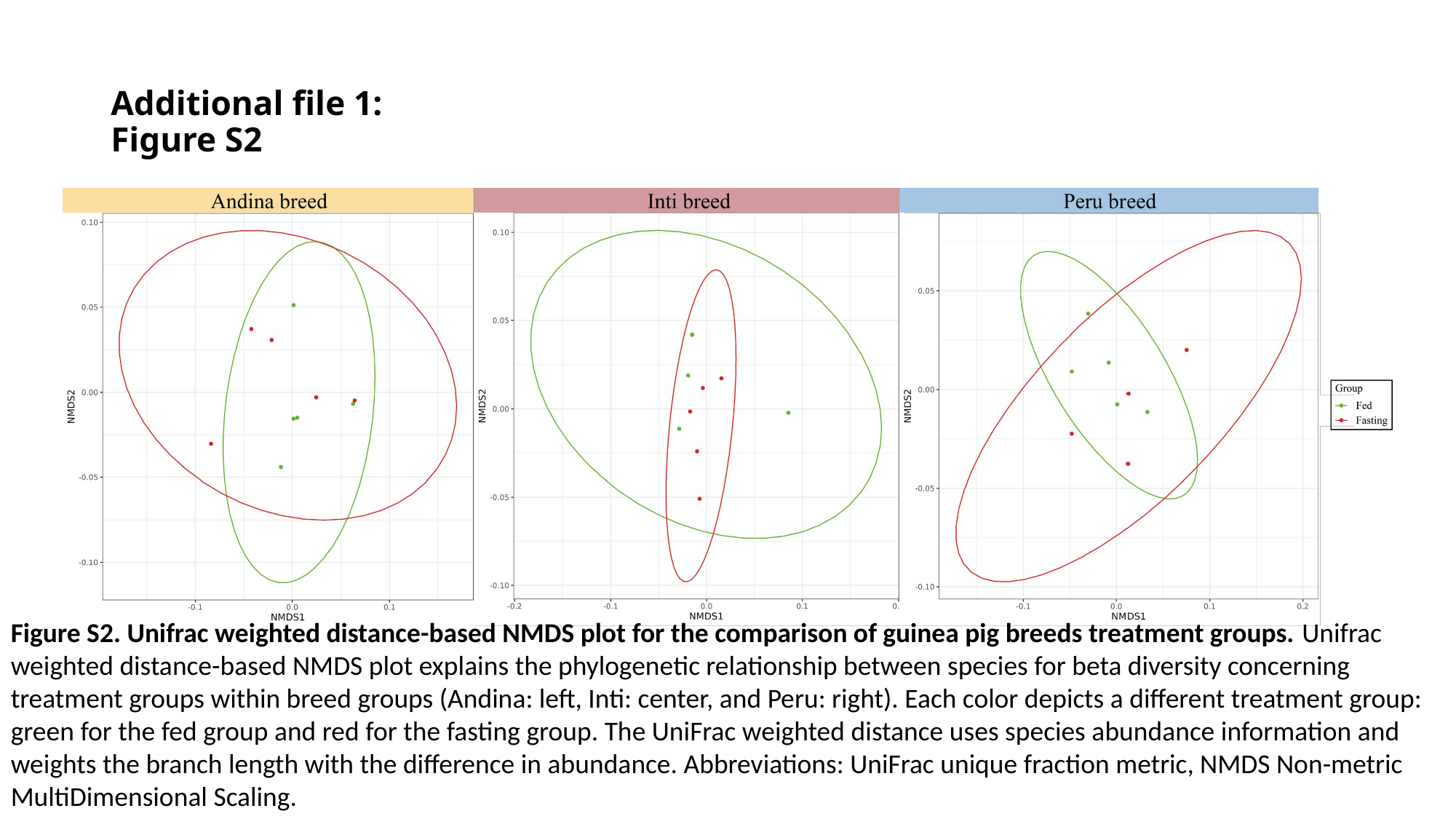

# Additional file 1: Figure S2
Figure S2. Unifrac weighted distance-based NMDS plot for the comparison of guinea pig breeds treatment groups. Unifrac weighted distance-based NMDS plot explains the phylogenetic relationship between species for beta diversity concerning treatment groups within breed groups (Andina: left, Inti: center, and Peru: right). Each color depicts a different treatment group: green for the fed group and red for the fasting group. The UniFrac weighted distance uses species abundance information and weights the branch length with the difference in abundance. Abbreviations: UniFrac unique fraction metric, NMDS Non-metric MultiDimensional Scaling.

## Slide 3
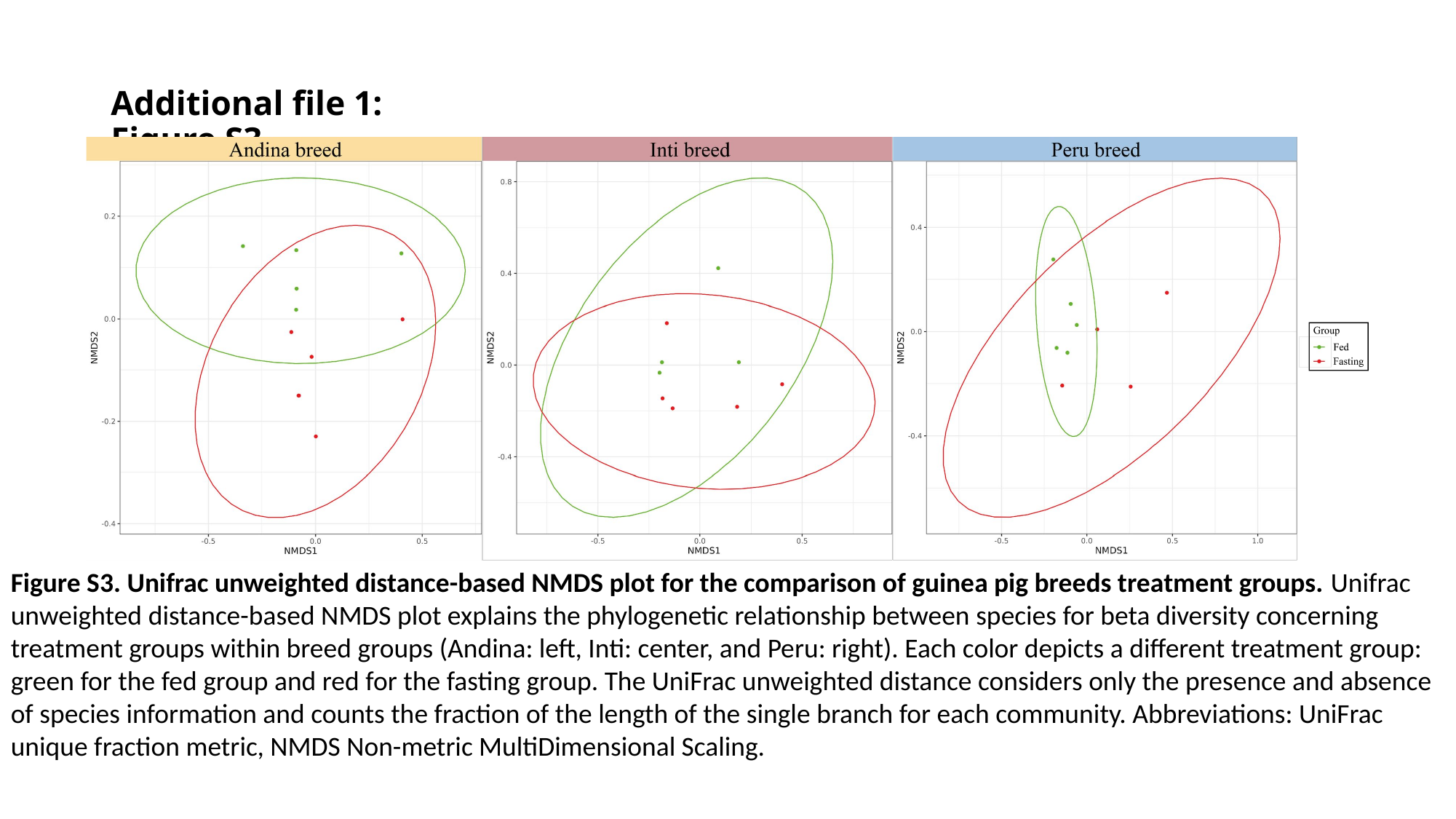

# Additional file 1: Figure S3
Figure S3. Unifrac unweighted distance-based NMDS plot for the comparison of guinea pig breeds treatment groups. Unifrac unweighted distance-based NMDS plot explains the phylogenetic relationship between species for beta diversity concerning treatment groups within breed groups (Andina: left, Inti: center, and Peru: right). Each color depicts a different treatment group: green for the fed group and red for the fasting group. The UniFrac unweighted distance considers only the presence and absence of species information and counts the fraction of the length of the single branch for each community. Abbreviations: UniFrac unique fraction metric, NMDS Non-metric MultiDimensional Scaling.

## Slide 4
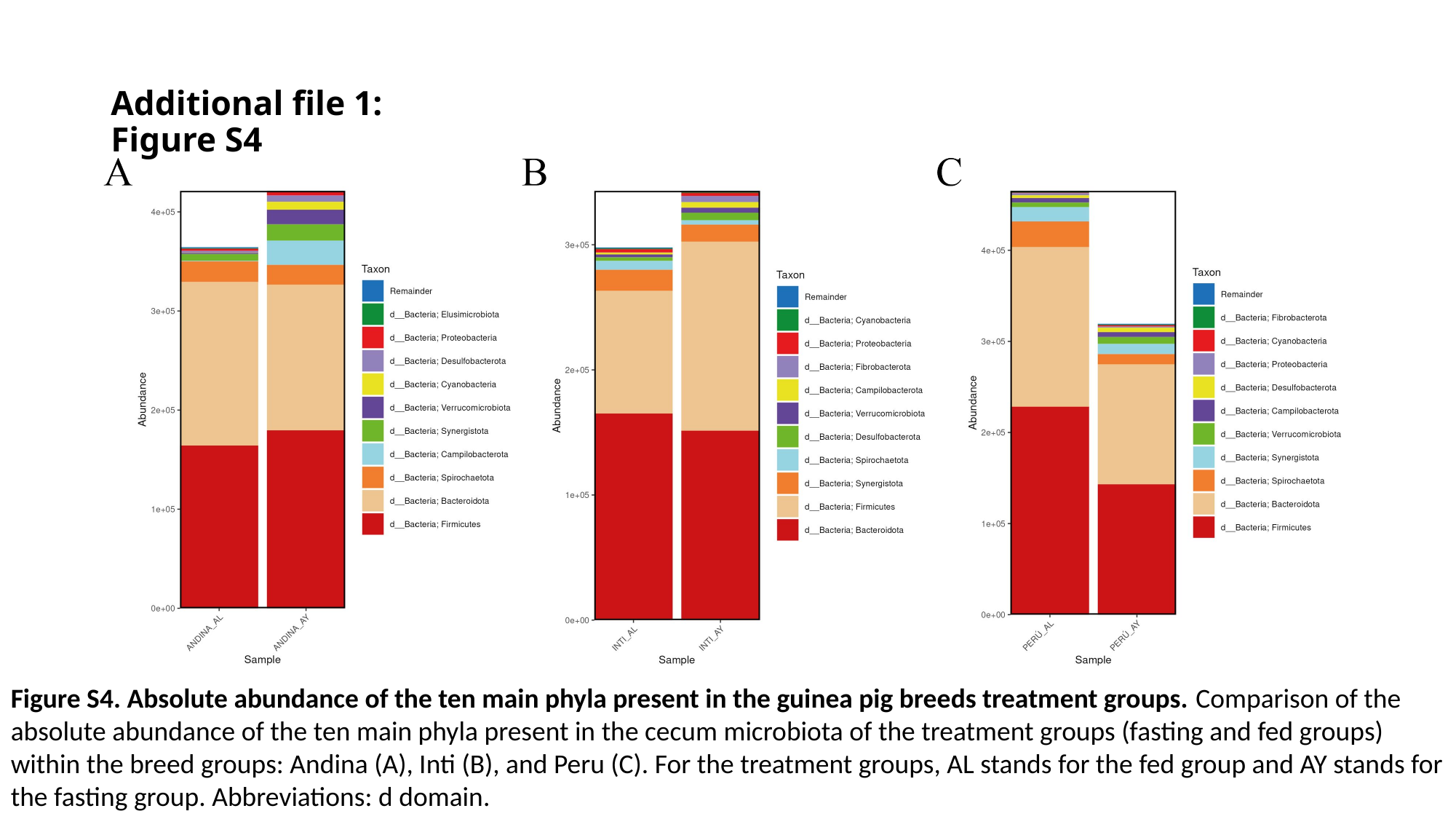

# Additional file 1: Figure S4
Figure S4. Absolute abundance of the ten main phyla present in the guinea pig breeds treatment groups. Comparison of the absolute abundance of the ten main phyla present in the cecum microbiota of the treatment groups (fasting and fed groups) within the breed groups: Andina (A), Inti (B), and Peru (C). For the treatment groups, AL stands for the fed group and AY stands for the fasting group. Abbreviations: d domain.

## Slide 5
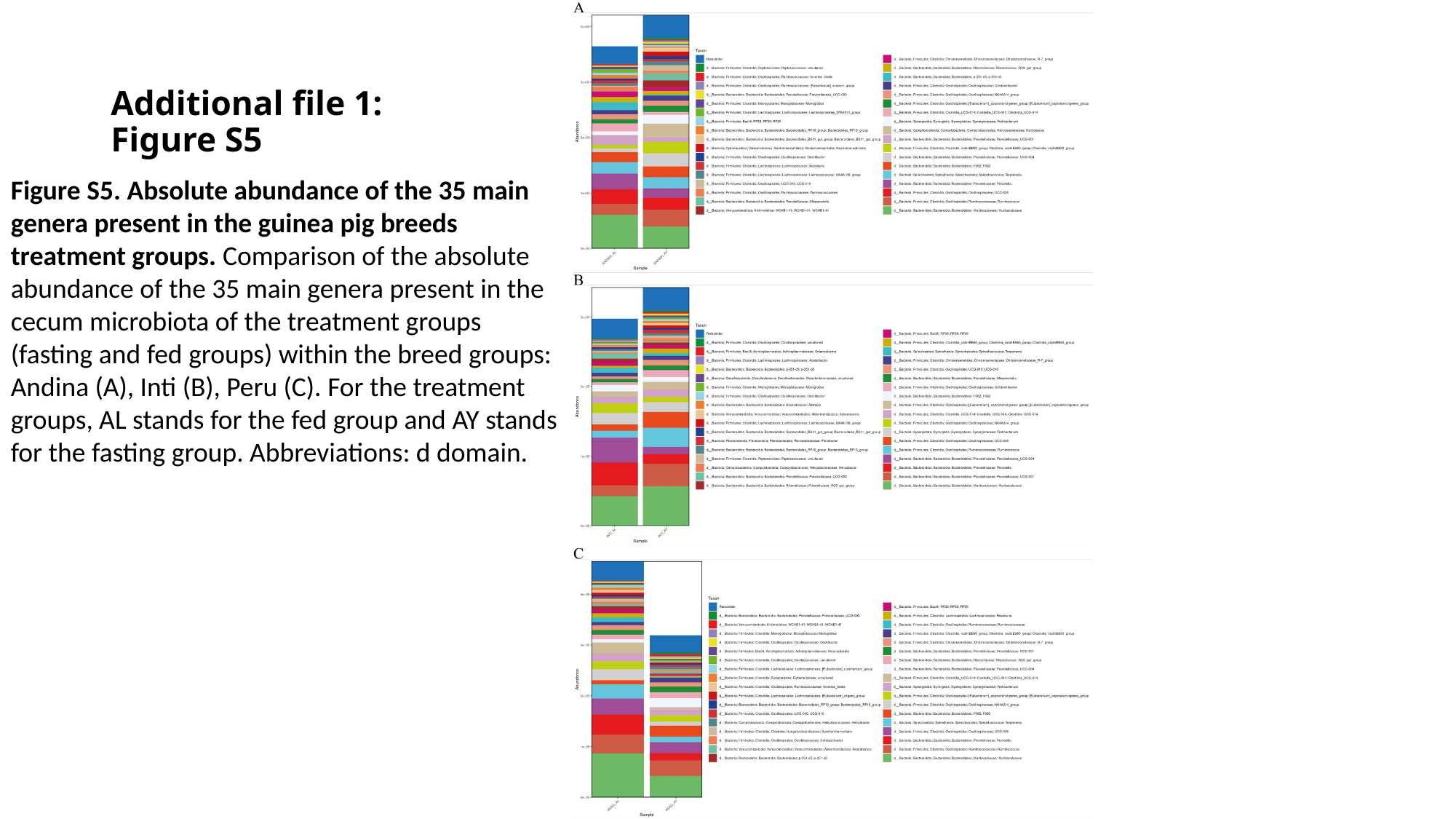

# Additional file 1: Figure S5
Figure S5. Absolute abundance of the 35 main genera present in the guinea pig breeds treatment groups. Comparison of the absolute abundance of the 35 main genera present in the cecum microbiota of the treatment groups (fasting and fed groups) within the breed groups: Andina (A), Inti (B), Peru (C). For the treatment groups, AL stands for the fed group and AY stands for the fasting group. Abbreviations: d domain.

## Slide 6
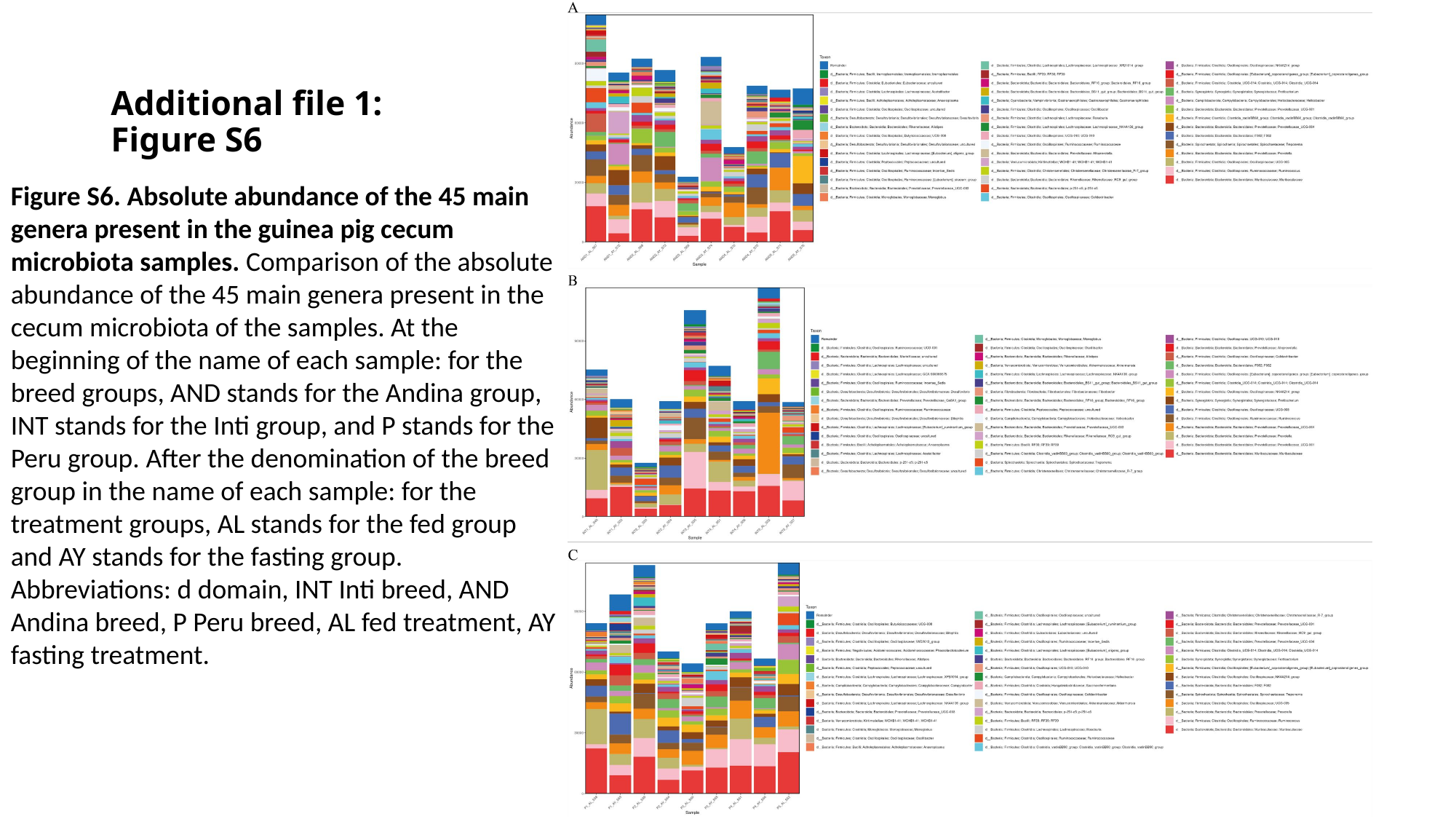

# Additional file 1: Figure S6
Figure S6. Absolute abundance of the 45 main genera present in the guinea pig cecum microbiota samples. Comparison of the absolute abundance of the 45 main genera present in the cecum microbiota of the samples. At the beginning of the name of each sample: for the breed groups, AND stands for the Andina group, INT stands for the Inti group, and P stands for the Peru group. After the denomination of the breed group in the name of each sample: for the treatment groups, AL stands for the fed group and AY stands for the fasting group. Abbreviations: d domain, INT Inti breed, AND Andina breed, P Peru breed, AL fed treatment, AY fasting treatment.
