# Supplementary material for: Comparative analysis of fasting effects on the cecum microbiome in three guinea pig breeds: Andina, Inti, and Peru
Source: Front Microbiol. 2023 Dec 20;14:1283738. doi: 10.3389/fmicb.2023.1283738 (PMC10761435; doi:10.3389/fmicb.2023.1283738)
Supplement: Supplementary file 3 [file Data_Sheet_2.DOCX]

**Additional file 2: Table S1.** **Alpha Diversity Analysis – Significance test of ANOVA for Shannon and Chao1 indices for Andina-Treatment groups.**

| ANOVA | | | | | | |
| --- | --- | --- | --- | --- | --- | --- |
| Summary (aov. Shannon) | Df | Sum-Sq | Mean-Sq | F-value | Pr(>F) | sig |
| Andina-Treatment | 1 | 0.0015 | 0.00154 | 0.02 | 0.891 |  |
| Residuals | 8 | 0.6191 | 0.07738 |  |  |  |
| Summary (aov. Chao1) |  |  |  |  |  |  |
| Andina-Treatment | 1 | 3572 | 3572 | 0.603 | 0.46 |  |
| Residuals | 8 | 47406 | 5926 |  |  |  |

^Abbreviations: aov Analysis of variance, Df: Degrees of freedom, Sum-Sq Sum of squares, Mean-Sq Sum of the squares divided by the degrees of freedom, Pr(>F) p-value of the F statistic, sig significance. Significance codes: 0 ‘***’ 0.001 ‘**’ 0.01 ‘*’ 0.05 ‘.’ 0.1 ‘ ’ 1^

**Additional file 2: Table S2**. **Alpha Diversity Analysis for index Shannon for Andina-Treatment groups.**

| Shannon | | | | | |
| --- | --- | --- | --- | --- | --- |
| Andina-Treatment | diff | lwr | upr | p | sig |
| ANDINA-fasting vs. ANDINA-fed | 0.02484823 | -0.3808598 | 0.4305563 | 0.8911761 |  |

^Tukey’s honest significance test of our ANOVA - Tukey multiple comparisons of means - 95% family-wise confidence level for Andina-Treatment groups. Abbreviations: diff mean difference between the groups, lwr lower-end point of the interval, upr upper-end point of the interval, p p-value, sig significance. Significance codes: 0 ‘***’ 0.001 ‘**’ 0.01 ‘*’ 0.05 ‘.’ 0.1 ‘ ’ 1^

**Additional file 2:Table S3**. **Analysis of Alpha Diversity for index Chao1 for Andina-Treatment groups.**

| Chao1 | | | | | |
| --- | --- | --- | --- | --- | --- |
| Andina-Treatment | diff | lwr | upr | p | sig |
| ANDINA-fasting vs. ANDINA-fed | 37.8 | -74.46949 | 150.0695 | 0.4598449 |  |

^Tukey's honest significance test of our ANOVA - Tukey multiple comparisons of means - 95% family-wise confidence level for Andina-Treatment groups. Abbreviations: diff mean difference between the groups, lwr lower-end point of the interval, upr upper-end point of the interval, p p-value, sig significance. Significance codes: 0 ‘***’ 0.001 ‘**’ 0.01 ‘*’ 0.05 ‘.’ 0.1 ‘ ’ 1^

**Additional file 2: Table S4**. **Alpha Diversity Analysis – Significance test of ANOVA for Shannon and Chao1 indices for Inti-Treatment groups.**

| ANOVA | | | | | | |
| --- | --- | --- | --- | --- | --- | --- |
| Summary (aov. Shannon) | Df | Sum-Sq | Mean-Sq | F-value | Pr(>F) | sig |
| Inti-Treatment | 1 | 0.2065 | 0.20647 | 3.721 | 0.0951 | **.** |
| Residuals | 7 | 0.3884 | 0.05549 |  |  |  |
| Summary (aov. Chao1) |  |  |  |  |  |  |
| Inti-Treatment | 1 | 2472 | 2472 | 0.545 | 0.484 |  |
| Residuals | 7 | 31720 | 4531 |  |  |  |

Abbreviations: aov Analysis of variance, Df: Degrees of freedom, Sum-Sq Sum of squares, Mean-Sq Sum of the squares divided by the degrees of freedom, Pr(>F) p-value of the F statistic, sig significance. Significance codes: 0 ‘***’ 0.001 ‘**’ 0.01 ‘*’ 0.05 ‘.’ 0.1 ‘ ’ 1

**Additional file 2: Table S5.** **Alpha Diversity Analysis for index Shannon for Inti-Treatment groups.**

| Shannon | | | | | |
| --- | --- | --- | --- | --- | --- |
| Inti-Treatment | diff | lwr | upr | p | sig |
| Inti-fasting vs. Inti-fed | 0.304816 | -0.06884 | 0.678471 | 0.095064 |  |

^Tukey’s honest significance test of our ANOVA - Tukey multiple comparisons of means - 95% family-wise confidence level for Inti-Treatment groups. Abbreviations: diff mean difference between the groups, lwr lower-end point of the interval, upr upper-end point of the interval, p p-value, sig significance. Significance codes: 0 ‘***’ 0.001 ‘**’ 0.01 ‘*’ 0.05 ‘.’ 0.1 ‘ ’ 1^

**Additional file 2: Table S6**. **Analysis of Alpha Diversity for index Chao1 for Inti-Treatment groups**

| Chao1 | | | | | |
| --- | --- | --- | --- | --- | --- |
| Inti-Treatment | diff | lwr | upr | p | sig |
| Inti-fasting vs. Inti-fed | 33.35 | -73.4289 | 140.1289 | 0.484208 |  |

^Tukey's honest significance test of our ANOVA - Tukey multiple comparisons of means - 95% family-wise confidence level for Inti-Treatment groups. Abbreviations: diff mean difference between the groups, lwr lower-end point of the interval, upr upper-end point of the interval, p p-value, sig significance. Significance codes: 0 ‘***’ 0.001 ‘**’ 0.01 ‘*’ 0.05 ‘.’ 0.1 ‘ ’ 1^

**Additional file 2: Table S7**. **Alpha Diversity Analysis – Significance test of ANOVA for Shannon and Chao1 indices for Peru-Treatment groups.**

| ANOVA | | | | | | |
| --- | --- | --- | --- | --- | --- | --- |
| Summary (aov. Shannon) | Df | Sum-Sq | Mean-Sq | F-value | Pr(>F) | sig |
| Peru-Treatment | 1 | 0.0006 | 0.00057 | 0.005 | 0.943 |  |
| Residuals | 7 | 0.7393 | 0.10562 |  |  |  |
| Summary (aov. Chao1) |  |  |  |  |  |  |
| Peru-Treatment | 1 | 7271 | 7271 | 0.858 | 0.385 |  |
| Residuals | 7 | 59327 | 8475 |  |  |  |

^Abbreviations: aov Analysis of variance, Df: Degrees of freedom, Sum-Sq Sum of squares, Mean-Sq Sum of the squares divided by the degrees of freedom, Pr(>F) p-value of the F statistic, sig significance. Significance codes: 0 ‘***’ 0.001 ‘**’ 0.01 ‘*’ 0.05 ‘.’ 0.1 ‘ ’ 1^

**Additional file 2: Table S8.** **Alpha Diversity Analysis for index Shannon for Peru-Treatment groups.**

| Shannon | | | | | |
| --- | --- | --- | --- | --- | --- |
| Peru-Treatment | diff | lwr | upr | p | sig |
| Peru-fasting vs. Peru-fed | -0.01607 | -0.53158 | 0.499442 | 0.943314 |  |

^Tukey’s honest significance test of our ANOVA - Tukey multiple comparisons of means - 95% family-wise confidence level for Peru-Treatment groups. Abbreviations: diff mean difference between the groups, lwr lower-end point of the interval, upr upper-end point of the interval, p p-value, sig significance. Significance codes: 0 ‘***’ 0.001 ‘**’ 0.01 ‘*’ 0.05 ‘.’ 0.1 ‘ ’ 1^

**Additional file 2: Table S9**. **Analysis of Alpha Diversity for index Chao1 for Peru-Treatment groups.**

| Chao1 | | | | | |
| --- | --- | --- | --- | --- | --- |
| Peru-Treatment | diff | lwr | upr | p | sig |
| Peru-fasting vs. Peru-fed | -57.2 | -203.231 | 88.83093 | 0.385153 |  |

^Tukey's honest significance test of our ANOVA - Tukey multiple comparisons of means - 95% family-wise confidence level for Peru-Treatment groups. Abbreviations: diff mean difference between the groups, lwr lower-end point of the interval, upr upper-end point of the interval, p p-value, sig significance. Significance codes: 0 ‘***’ 0.001 ‘**’ 0.01 ‘*’ 0.05 ‘.’ 0.1 ‘ ’ 1^

**Additional file 2: Table S10**. **Statistical analysis of beta diversity for the comparison of Andina-Treatment groups.**

| Beta Diversity | | | | | | | | |
| --- | --- | --- | --- | --- | --- | --- | --- | --- |
| Andina-Treatment | Df | SumsOfSqs | F.Model | R2 | p-value | sig | p.adjusted | sig |
| Andina-fed vs. Andina-fasting | 1 | 0.2572464 | 1.231554 | 0.133407 | 0.088 |  | 0.088 |  |

^Pairwise Permanova, considering the Unweighted Unifrac method in the calculation, and with the Bonferroni correction for the comparison of Andina-Treatment groups. The F.Model value was obtained by the ratio of the Mean Squares (which are the Sum of squares divided by their degrees of freedom). Abbreviations: Df Degrees of freedom, SumsOfSqs Sums of squares, F.Model F.Model value, R2 R squared, sig significance. Significance codes: 0 ‘***’ 0.001 ‘**’ 0.01 ‘*’ 0.05 ‘.’ 0.1 ‘ ’ 1^

**Additional file 2: Table S11**. **Statistical analysis of beta diversity for the comparison of Inti-Treatment groups.**

| Beta Diversity | | | | | | | | |
| --- | --- | --- | --- | --- | --- | --- | --- | --- |
| Inti-Treatment | Df | SumsOfSqs | F.Model | R2 | p-value | sig | p.adjusted | sig |
| Inti-fed vs. Inti-fasting | 1 | 0.002978 | 0.895404 | 0.113408 | 0.568 |  | 0.568 |  |

^Pairwise Permanova, considering the weighted Unifrac method in the calculation, and with the Bonferroni correction for the comparison of Inti-Treatment groups. The F.Model value was obtained by the ratio of the Mean Squares (which are the Sum of squares divided by their degrees of freedom). Abbreviations: Df Degrees of freedom, SumsOfSqs Sums of squares, F.Model F.Model value, R2 R squared, sig significance. Significance codes: 0 ‘***’ 0.001 ‘**’ 0.01 ‘*’ 0.05 ‘.’ 0.1 ‘ ’ 1^

**Additional file 2: Table S12**. **Statistical analysis of beta diversity for the comparison of Peru-Treatment groups**.

| Beta Diversity | | | | | | | | |
| --- | --- | --- | --- | --- | --- | --- | --- | --- |
| Peru-Treatment | Df | SumsOfSqs | F.Model | R2 | p-value | sig | p.adjusted | sig |
| Peru-fed vs. Peru fasting | 1 | 0.241416 | 1.345009 | 0.161175 | 0.049 | ***** | 0.049 | ***** |

^Pairwise Permanova, considering the Unweighted Unifrac method in the calculation, and with the Bonferroni correction for the comparison of Peru-Treatment groups. The F.Model value was obtained by the ratio of the Mean Squares (which are the Sum of squares divided by their degrees of freedom). Abbreviations: Df Degrees of freedom, SumsOfSqs Sums of squares, F.Model F.Model value, R2 R squared, sig significance. Significance codes: 0 ‘***’ 0.001 ‘**’ 0.01 ‘*’ 0.05 ‘.’ 0.1 ‘ ’ 1^
