# Supplementary material for: Comparative analysis of fasting effects on the cecum microbiome in three guinea pig breeds: Andina, Inti, and Peru
Source: Front Microbiol. 2023 Dec 20;14:1283738. doi: 10.3389/fmicb.2023.1283738 (PMC10761435; doi:10.3389/fmicb.2023.1283738)
Supplement: Supplementary file 7 [file Data_Sheet_6.DOCX]

**Additional File 7: Contents of the Additional Files 3-6**

**Contents of the worksheet from the Additional File 3 Taxa abundance**

**1-Rel_allgenera_samplesA-t:** Relative abundance of all genera found in the Andina breed samples for the treatments: AL (fed) and AY (fasting). The whole taxonomy information for each genus was detailed in the first six columns from left to right: Kingdom, Phylum, Class, Order, Family, and Genus. The following columns detailed the numeric values of the relative abundance of the genera for each Andina breed sample (sample for column).

**2-Rel_allgenera_A-t:** Relative abundance of all genera found in the Andina breed treatments: Andina-fed and Andina-fasting. The whole taxonomy information for each genus was detailed in the first six columns from left to right: Kingdom, Phylum, Class, Order, Family, and Genus. The following columns detailed numeric values of the relative abundance of the genera for each Andina breed treatment (Andina-fed and Andina-fasting).

**3-Rel_45genera_A-t_sample:** Relative abundance of the 45 most abundant genera found in the Andina breed samples for the treatments: AL (fed) and AY (fasting). The whole taxonomy information for each genus was detailed in the first six columns from left to right: Kingdom, Phylum, Class, Order, Family, and Genus. The following columns detailed the numeric values of the relative abundance of the 45 most abundant genera for each Andina breed sample (sample for column).

**4-Rel_35genera_A-t:** Relative abundance of the 35 most abundant genera found in the Andina breed treatments: Andina-fed and Andina-fasting. The whole taxonomy information for each genus was detailed in the first six columns from left to right: Kingdom, Phylum, Class, Order, Family, and Genus. The following columns detailed numeric values of the relative abundance of the 35 most abundant genera for each Andina breed treatment (Andina-fed and Andina-fasting).

**5-Rel_allphyla_A-t:** Relative abundance of all phyla found in the Andina breed treatments: Andina-fed and Andina-fasting. The whole taxonomy information for each phyla was detailed in the first two columns from left to right: Kingdom and Phylum. The following columns detailed numeric values of the relative abundance of all the phyla for each Andina breed treatment (Andina-fed and Andina-fasting).

**6-Rel_10phyla_A-t:** Relative abundance of the ten most abundant phyla found in the Andina breed treatments: Andina-fed and Andina-fasting. The whole taxonomy information for each phyla was detailed in the first two columns from left to right: Kingdom and Phylum. The following columns detailed numeric values of the relative abundance of the ten most abundant phyla for each Andina breed treatment (Andina-fed and Andina-fasting).

**7-Rel_allgenera_samplesI-t:** Relative abundance of all genera found in the Inti breed samples for the treatments: AL (fed) and AY (fasting). The whole taxonomy information for each genus was detailed in the first six columns from left to right: Kingdom, Phylum, Class, Order, Family, and Genus. The following columns detailed the numeric values of the relative abundance of the genera for each Inti breed sample (sample for column).

**8-Rel_allgenera_I-t:** Relative abundance of all genera found in the Andina breed treatments: Inti-fed and Inti-fasting. The whole taxonomy information for each genus was detailed in the first six columns from left to right: Kingdom, Phylum, Class, Order, Family, and Genus. The following columns detailed numeric values of the relative abundance of the genera for each Inti breed treatment (Inti-fed and Inti-fasting).

**9-Rel_45genera_I-t_sample:** Relative abundance of the 45 most abundant genera found in the Inti breed samples for the treatments: AL (fed) and AY (fasting). The whole taxonomy information for each genus was detailed in the first six columns from left to right: Kingdom, Phylum, Class, Order, Family, and Genus. The following columns detailed the numeric values of the relative abundance of the 45 most abundant genera for each Inti breed sample (sample for column).

**10-Rel_35genera_I-t:** Relative abundance of the 35 most abundant genera found in the Inti breed treatments: Inti-fed and Inti-fasting. The whole taxonomy information for each genus was detailed in the first six columns from left to right: Kingdom, Phylum, Class, Order, Family, and Genus. The following columns detailed numeric values of the relative abundance of the 35 most abundant genera for each Inti breed treatment (Inti-fed and Inti-fasting).

**11-Rel_allphyla_I-t:** Relative abundance of all phyla found in the Inti breed treatments: Inti-fed and Inti-fasting. The whole taxonomy information for each phyla was detailed in the first two columns from left to right: Kingdom and Phylum. The following columns detailed numeric values of the relative abundance of all the phyla for each Inti breed treatment (Inti-fed and Inti-fasting).

**12-Rel_10phyla_I-t:** Relative abundance of the ten most abundant phyla found in the Inti breed treatments: Inti-fed and Inti-fasting. The whole taxonomy information for each phyla was detailed in the first two columns from left to right: Kingdom and Phylum. The following columns detailed numeric values of the relative abundance of the ten most abundant phyla for each Inti breed treatment (Inti-fed and Inti-fasting).

**13-Rel_allgenera_samplesP-t:** Relative abundance of all genera found in the Peru breed samples for the treatments: AL (fed) and AY (fasting). The whole taxonomy information for each genus was detailed in the first six columns from left to right: Kingdom, Phylum, Class, Order, Family, and Genus. The following columns detailed the numeric values of the relative abundance of the genera for each Peru breed sample (sample for column).

**14-Rel_allgenera_P-t:** Relative abundance of all genera found in the Andina breed treatments: Peru-fed and Peru-fasting. The whole taxonomy information for each genus was detailed in the first six columns from left to right: Kingdom, Phylum, Class, Order, Family, and Genus. The following columns detailed numeric values of the relative abundance of the genera for each Peru breed treatment (Peru-fed and Peru-fasting).

**15-Rel_45genera_P-t_sample:** Relative abundance of the 45 most abundant genera found in the Peru breed samples for the treatments: AL (fed) and AY (fasting). The whole taxonomy information for each genus was detailed in the first six columns from left to right: Kingdom, Phylum, Class, Order, Family, and Genus. The following columns detailed the numeric values of the relative abundance of the 45 most abundant genera for each Peru breed sample (sample for column).

**16-Rel_35genera_P-t:** Relative abundance of the 35 most abundant genera found in the Peru breed treatments: Peru-fed and Peru-fasting. The whole taxonomy information for each genus was detailed in the first six columns from left to right: Kingdom, Phylum, Class, Order, Family, and Genus. The following columns detailed numeric values of the relative abundance of the 35 most abundant genera for each Peru breed treatment (Peru-fed and Peru-fasting).

**17-Rel_allphyla_samplesP-t:** Relative abundance of all phyla found in the Peru breed treatments: Peru-fed and Peru-fasting. The whole taxonomy information for each phyla was detailed in the first two columns from left to right: Kingdom and Phylum. The following columns detailed numeric values of the relative abundance of all the phyla for each Peru breed treatment (Peru-fed and Peru-fasting).

**18-Rel_10phyla_P-t:** Relative abundance of the ten most abundant phyla found in the Peru breed treatments: Peru-fed and Peru-fasting. The whole taxonomy information for each phyla was detailed in the first two columns from left to right: Kingdom and Phylum. The following columns detailed numeric values of the relative abundance of the ten most abundant phyla for each Peru breed treatment (Peru-fed and Peru-fasting).

**Contents of the worksheet from the Additional File 4 LDA**

**1-LDA_ANDINA:** Linear discriminant analysis (LDA) effect size (LEfSe) comparison of the taxa present in the Andina breed treatment groups. LefSe comparison of differentially abundant bacterial taxa between the treatment groups (fed and fasting groups) of the Andina breed. One red asterisk (p<0.05) and two red asterisks (p<0.01) denote taxa with statistically significant differences between the abundances of the Andina breed treatment groups. Abbreviations: LDA Linear discriminant analysis score, P. unadj. P-value unadjusted, P. adj P-value adjusted.

**2-LDA_INTI:** Linear discriminant analysis (LDA) effect size (LEfSe) comparison of the taxa present in the Inti breed treatment groups. LefSe comparison of differentially abundant bacterial taxa between the treatment groups (fed and fasting groups) of the Inti breed. One red asterisk (p<0.05) and two red asterisks (p<0.01) denote taxa with statistically significant differences between the abundances of the Inti breed treatment groups. Abbreviations: LDA Linear discriminant analysis score, P. unadj. P-value unadjusted, P. adj P-value adjusted.

**3-LDA_PERU:** Linear discriminant analysis (LDA) effect size (LEfSe) comparison of the taxa present in the Peru breed treatment groups. LefSe comparison of differentially abundant bacterial taxa between the treatment groups (fed and fasting groups) of the Peru breed. One red asterisk (p<0.05) and two red asterisks (p<0.01) denote taxa with statistically significant differences between the abundances of the Peru breed treatment groups. Abbreviations: LDA Linear discriminant analysis score, P. unadj. P-value unadjusted, P. adj P-value adjusted.

**Contents of the worksheet from the Additional File 5 Heat tree**

**1-diff_table_wilcoxon_ANDINA:** Data of the differential heat tree comparison between the taxa of the Andina treatment groups (Andina-fed and Andina-fasting). Abbreviations: p phylum, c class, o order, f family, g genus, s species.

**2-diff_table_wilcoxon_INTI:** Data of the differential heat tree comparison between the taxa of the Inti treatment groups (Inti-fed and Inti-fasting). Abbreviations: p phylum, c class, o order, f family, g genus, s species.

**3-diff_table_wilcoxon_PERU:** Data of the differential heat tree comparison between the taxa of the Peru treatment groups (Peru-fed and Peru-fasting). Abbreviations: p phylum, c class, o order, f family, g genus, s species.

**Contents of the worksheet from the Additional File 6 Venn chart**

**1-Venn_ANDINA:** List of the genera identified for the Andina breed treatment groups (Andina-fed and Andina fasting). Some genera may have the same name but different amplicon sequence variants (ASVs).

**2-Subsets_ANDINA:** List of the unique and shared genera identified for the Andina breed treatment groups (Andina-fed and Andina-fasting). The first column (only Andina fasting) from left to right details the genera that only was found in the Andina-fasting group. The second column details the genera that were shared between the Andina-fasting and Andina-fed groups. The third column (only Andina fed) details the genera that only was found in the Andina-fed group. Some genera may have the same name but different amplicon sequence variants (ASVs).

**3-Venn_INTI:** List of the genera identified for the Inti breed treatment groups (Inti-fed and Inti-fasting). Some genera may have the same name but different amplicon sequence variants (ASVs).

**4-Subsets_INTI:** List of the unique and shared genera identified for the Inti breed treatment groups (Inti-fed and Inti-fasting). The first column (only Inti fasting) from left to right details the genera that only was found in the Inti-fasting group. The second column details the genera that were shared between the Inti-fasting and Inti-fed groups. The third column (only Inti fed) details the genera that only was found in the Inti-fed group. Some genera may have the same name but different amplicon sequence variants (ASVs).

**5-Venn_PERU:** List of the genera identified for the Peru breed treatment groups (Peru-fed and Peru-fasting). Some genera may have the same name but different amplicon sequence variants (ASVs).

**6-Subsets_PERU:** List of the unique and shared genera identified for the Peru breed treatment groups (Peru-fed and Peru-fasting). The first column (only Peru fasting) from left to right details the genera that only was found in the Peru-fasting group. The second column details the genera that were shared between the Peru-fasting and Peru-fed groups. The third column (only Peru fed) details the genera that only was found in the Peru-fed group. Some genera may have the same name but different amplicon sequence variants (ASVs).
